# Reinforcement learning for solution updating in Artificial Bee Colony

**DOI:** 10.1371/journal.pone.0200738

**Published:** 2018-07-17

**Authors:** Suthida Fairee, Santitham Prom-On, Booncharoen Sirinaovakul

**Affiliations:** Department of Computer Engineering, King Mongkut’s University of Technology Thonburi, Bangkok, Thailand; Southwest University, CHINA

## Abstract

In the Artificial Bee Colony (ABC) algorithm, the employed bee and the onlooker bee phase involve updating the candidate solutions by changing a value in one dimension, dubbed one-dimension update process. For some problems which the number of dimensions is very high, the one-dimension update process can cause the solution quality and convergence speed drop. This paper proposes a new algorithm, using reinforcement learning for solution updating in ABC algorithm, called R-ABC. After updating a solution by an employed bee, the new solution results in positive or negative reinforcement applied to the solution dimensions in the onlooker bee phase. Positive reinforcement is given when the candidate solution from the employed bee phase provides a better fitness value. The more often a dimension provides a better fitness value when changed, the higher the value of update becomes in the onlooker bee phase. Conversely, negative reinforcement is given when the candidate solution does not provide a better fitness value. The performance of the proposed algorithm is assessed on eight basic numerical benchmark functions in four categories with 100, 500, 700, and 900 dimensions, seven CEC2005’s shifted functions with 100, 500, 700, and 900 dimensions, and six CEC2014’s hybrid functions with 100 dimensions. The results show that the proposed algorithm provides solutions which are significantly better than all other algorithms for all tested dimensions on basic benchmark functions. The number of solutions provided by the R-ABC algorithm which are significantly better than those of other algorithms increases when the number of dimensions increases on the CEC2005’s shifted functions. The R-ABC algorithm is at least comparable to the state-of-the-art ABC variants on the CEC2014’s hybrid functions.

## Introduction

The Artificial Bee Colony (ABC) algorithm [1] is a meta-heuristic optimization algorithm based on Swarm Intelligence. A swarm system comprises simple agents which communicate with other agents and their environment. By targeting the same goal, agents complete the swarm’s task without any control unit. In the Artificial Bee Colony algorithm, the agents’ goal is to find the best food source.

Food sources represent a set of feasible solutions in a multidimensional search space, and each agent simulates a bee. A solution is composed of optimization parameters. The number of dimensions specifies the number of optimization parameters. For example, a 10-dimension solution is composed of 10 optimization parameters. The larger the number of dimensions, the more complicated a problem is.

In ABC, agents are categorized by their functions into three groups, which are employed bees, onlooker bees, and scout bees. Each employed bee is initially associated with a random food source. In each iteration, employed bees explore new food sources near the current ones. After collecting nectar, each employed bee evaluates how good the food source is, and it moves to the new food source only if the bee determines that the new source is better. The employed bees also share the information with onlooker bees. Each onlooker bee decides to establish a rich food source based on the information provided by the employed bees. The higher the quality of a food source, the higher the probability the food source is selected by onlooker bees. Each onlooker bee then finds a new food source around the selected food source and moves to the new food source if it finds that the new food source is better. In a predetermined number of iterations, if a better food source cannot be found, employed bees associated with a food source may turn into scout bees and explore new food sources in a new area of the search space.

There are both similarities and differences in finding new food sources by employed bees, onlooker bees, and scout bees. An employed bee finds a new food source close to the food source with which it is associated, irrespective of how good the current food source is, whereas an onlooker bee tends to find a new food source around a high quality one. An employed bee finds a new food source around the current food source by changing a value in one dimension, and so does an onlooker bee. After that, both employed bees and onlooker bees choose to stay with the better food sources, i.e. greedy selection. In contrast, scout bees will find new food sources in the search space by changing values in all dimensions and always move to the new ones, without considering their quality.

Each group of bees displays a different degree of explorative and exploitative behaviors. The explorative behavior of a search agent involves searching for a new food source in a large region of the search space to avoid a local optimum. On the other hand, the exploitative behavior involves searching for a better food source near the current food source. Comparing explorative and exploitative behaviors, the scout bees perform the highest degree of exploration while the onlooker bees perform the highest degree of exploitation. The scout bees’ explorative behavior enables the widest search range, i.e. their new food sources could be any food sources in the search space, possibly in an unknown area. The search ranges of the employed bees and onlooker bees, in contrast, are only in the area around the existing food sources. The onlooker bees’ exploitative behavior makes them likely to find the best quality food source because each onlooker bee tends to have a good quality food source in hand while it searches for a better one.

Inspired by a variant of Particle Swarm Optimization (PSO) called Bare Bones PSO [2], Gao, Chan, Huang, and Liu [3] introduced Bare Bones ABC (BABC) with three key modifications. First, onlooker bees use a Gaussian search equation to generate a new food source. Second, a new adaptation strategy is applied to the scaling factor of the search equation in the employed bee phase. Third, a fitness-based neighborhood mechanism was introduced. To update the current food source, an employed bee proportionally selects an individual food source based on the fitness values. The better fitness value a food source has, the more probability it will be selected. The three modifications increase the exploitation of the employed bee and onlooker bee phases. Kiran, Hakli, Gunduz, and Uguz [4] proposed the ABC algorithm with a variable search strategy (ABCVSS) using five different search equations and their individual counters. Each search equation plays a different role of searching. One of the search equations is the original ABC’s search equation. Two search equations are used to increase the diversity of the population. Another search equation is used to search around the global best food source, while the other search equation is used to search towards the mean of the population. The counter of a search equation indicates the number of the successful searches. The search equation selection is based on the values of the counters. The higher value of the counter a search equation has, the more probability it will be selected. The variable search strategy allows the ABC algorithm to learn which search equation provides good solutions for each problem. Cui, Li, Zhu, Lin, Wen, Lu, et al. [5] introduced an adaptive method for the population size (AMPS). The population size is adaptive according to the number of the successful searches and this population control strategy is used to balance the degree of exploitation and exploration adaptively. In [6], a ranking-based adaptive selection probability is used. Rather than the fitness values, the selection probability is calculated from the ranking of each food source and the success rate of the population. If the success rate is high, the exploitation is preferable to the exploration and an onlooker bee tends to select a good food source. On the other hand, if the success rate is low, the exploration is preferable to the exploitation.

Lately, Li, Cui, Fu, Wen, Lu, and Lu [7] introduced a gene recombination operator (GRO) at the end of each generation. As an extension, the GRO is used to generate a new food source from good food sources. It accelerates the convergence speed and increases the exploitation. However, the GRO is not efficient in the case of multimodal functions, because two good food sources used to generate a new food source might be located near two different local optimum food sources.

Like other optimization algorithms, the performance of the ABC algorithm in the aspect of solution quality drops when the number of dimensions increases. There have been many efforts to improve the solution quality of the ABC algorithm. One of the widely-used techniques is driving a new solution towards the best quality food source. Inspired by the PSO algorithm, Zhu and Kwong [8] proposed the Gbest-guided Artificial Bee Colony (GABC). In the GABC algorithm, a non-negative control parameter C was used to steer the new food source towards the best quality food source as the global best.

To accelerate driving towards the best solution, Banharnsakun, Achalakul, and Sirinaovakul [9] proposed the Best-so-far Artificial Bee Colony (BSF-ABC) which not only drives the food source towards the best food source but also updates values in all dimensions. There are two key modifications. First, BSF-ABC updates all dimensions of the food source towards the best-so-far solution rather than updating only one dimension towards a random food source. Because all dimensions are updated towards the best-so-far food source, the convergence speed is very high. Second, a scout bee discovers a new food source within an adjustable search area. The search area is largest during the early iterations and shrinks over time.

Akay and Karaboga [10] proposed a modified ABC (MABC) which updates more than one dimension with the controllable magnitude of the perturbation. They introduced two parameters, modification rate (*MR*) and scaling factor (*SF*). The modification rate controls the diversity of the food source update and the number of dimensions changed. The higher the modification rate is, the larger the number of dimensions that are changed. The other control parameter, scaling factor or SF, controls the magnitude of the food source update. Both control parameters are fixed and predefined.

Karaboga and Kaya [11] proposed a new version of ABC named Adaptive and Hybrid Artificial Bee Colony (aABC). The aABC algorithm drives a new food source towards the best food source using an arithmetic crossover operation and updates a food source within an adaptable magnitude of the perturbation. A new food source update in the onlooker bee phase was introduced with two new control parameters, crossover rate, and adaptivity coefficient. The crossover rate determines how much the new food source is like the current food source. The adaptivity coefficient controls how quickly a bee moves towards the random food source. The crossover with the global best solution improves the quality of the generated food sources resulting in quick convergence, while the adaptivity coefficient enables the magnitude of food source update to be adaptable. The lower the adaptivity coefficient, the larger the magnitude of an update.

However, real-world optimization problems in many areas tend to be high-dimensional, for example, in biology [12] and visualization [13]. The growing number of dimensions, from tens to hundreds or even thousands, makes optimization problems much more difficult because the search space exponentially expands as the number of dimensions increases [14]. Therefore, the time complexity increases.

It is obvious that updating a value in only one dimension at a time is not sufficient to solve a high-dimensional problem. When updating many dimensions, the algorithm should not change all of them within the same magnitude of perturbation for all dimensions because the distance between the current food source and the actual best food source might be different in each dimension. The problem causes the algorithm unable to converge to an optimal solution in the problem like the Dixon-Price function. The algorithm should learn to adapt the degree of perturbation for each dimension separately.

In this paper, we use reinforcement learning to improve the solution quality when finding the solution in a very high dimensional problem. The ABC algorithm has strong explorative behavior and weak exploitative behavior, so we aim to improve the exploitative behavior in the onlooker bee phase. Inspired by the foraging behavior of animals, we introduce the concept of the reinforcement vector. When an animal forages for food, it is more likely to keep moving in the same direction rather than turning to a different direction. The animal will often search for more food in the same area if it can find food, so called win-stay lose-shift strategy [15]. In the ABC algorithm, a bee moves in a direction by changing the value of a dimension. In the proposed algorithm, when an employed bee moves to a direction and finds a candidate food source, it does not share only the quality of its food source with onlooker bees but also the direction. The employed bees then update the reinforcement vector with positive or negative reinforcement. If an employed bee finds a better food source, positive reinforcement is given to the dimension. On the other hand, if the employed bee cannot find a better food source, negative reinforcement is given to the dimension. The idea is to driving onlooker bees’ positions with different magnitude of perturbation for each dimension according to the reinforcement vector. An onlooker bee changes the values of all dimensions, and it is likely to change the values of some dimensions more than those of others. Therefore, the algorithm will improve the exploitation performance of ABC when finding the solution in a very high dimensional problem.

## Reinforcement learning for solution updating

Modifying the original ABC algorithm [1,16], we use reinforcement learning for solution updating, introducing a new algorithm called R-ABC algorithm. In the *t*^th^ iteration, a population or a set of food sources is denoted by *X*^*t*^ consisting of *SN* food sources. The *i*^th^ food source of *X*^*t*^ is demoted by ${\bar{x}}^{t}{}_{i}$, *i* ∈ {1, 2, 3, …, *SN*}, as shown in Eq (1).

$$X^{t}=\left\{{\vec{x}}_{1}^{t},{\vec{x}}_{2}^{t},{\vec{x}}_{3}^{t},\ldots ,{\vec{x}}_{SN}^{t}\right\}$$ (1)

For a *D*-dimensional problem, each food source ${\bar{x}}^{t}{}_{i}$ has *D* optimization parameters. The *j*^th^ optimization parameter of ${\bar{x}}^{t}{}_{i}$ is denoted by *x*^*t*^_*i*,*j*_, *j* ∈ {1, 2, 3, …, *D*}, as shown in Eq (2).

$${\vec{x}}_{i}^{t}=\langle x_{i,1}^{t},x_{i,2}^{t},x_{i,3}^{t},\ldots ,x_{i,D}^{t}\rangle$$ (2)

The fitness value of ${\bar{x}}_{i}{}^{t}$ is denoted by $Fit({\bar{x}}_{i}{}^{t})$. The objective of the algorithm is to find the optimization parameters providing the minimum value of $Fit({\bar{x}}_{i}{}^{t})$, and the fitness value of ${\bar{x}}_{i}{}^{t}$ is defined as in Eq (3).

$$Fit({\vec{x}}_{i}^{t})=\{\begin{array}{cc} \frac{1}{1+f_{i}^{t}} & ,f_{i}^{t}\geq 0\\ 1+\left|f_{i}^{t}\right| & ,f_{i}^{t}<0 \end{array}$$ (3)

Where *f*_*i*_^*t*^ is the objective function value of the food source ${\bar{x}}_{i}{}^{t}$.

A reinforcement vector ${\bar{r}}^{t}$ is introduced for *X*^*t*^ in the R-ABC algorithm. For a *D*-dimensional problem, the reinforcement vector ${\bar{r}}^{t}$ is defined as in Eq (4).

$${\vec{r}}^{t}=\langle r_{1}^{t},r_{2}^{t},r_{3}^{t},\ldots ,r_{D}^{t}\rangle ,r_{j}^{t}\in \left[0,1\right],\sum_{j=1}^{D}r_{j}^{t}=1$$ (4)

**Fig 1:**
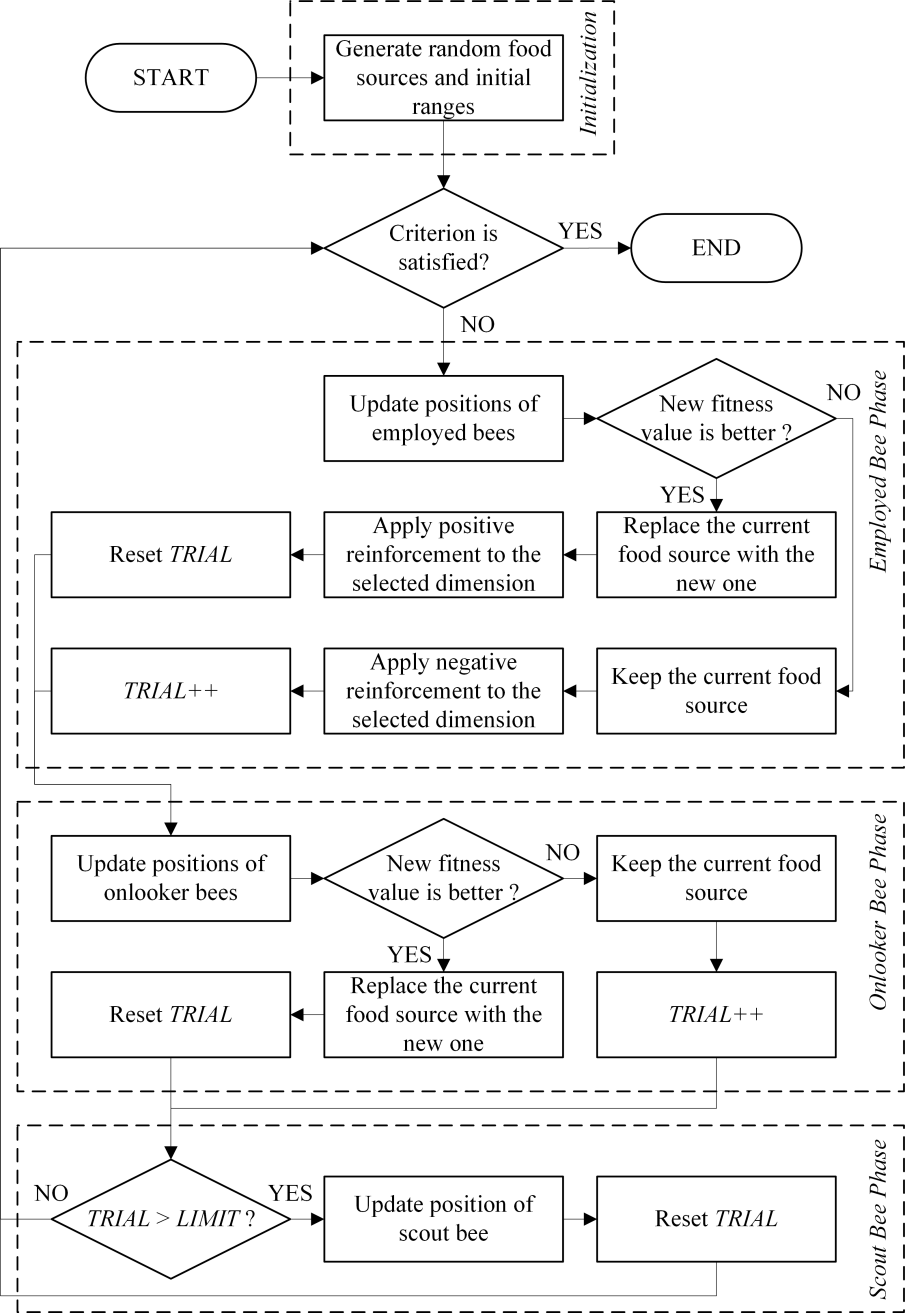
Flowchart of the R-ABC algorithm.

Fig 1 shows a flowchart of the R-ABC algorithm. The reinforcement value *r*^*t*^_*j*_ is used as the reinforcement for the *j*^th^ optimization parameters of all food sources in *X*^*t*^. In other words, the optimization parameters *x*^*t*^_*1*,*j*_, *x*^*t*^_*2*,*j*_, *x*^*t*^_*3*,*j*_, …, and *x*^*t*^_*SN*,*j*_ share the same reinforcement value (*r*^*t*^_*j*_).

For initialization, food sources are randomly generated as shown in Eq (5) and then employed bees are associated with food sources. The initial reinforcement *r*^*0*^_*j*_ is calculated from Eq (6).

$$x_{i,j}^{0}=x_{j}^{\min}+rand(0,1)\cdot \left(x_{j}^{\max}-x_{j}^{\min}\right)$$ (5)

$$r_{j}^{0}=\frac{1}{D}$$ (6)

Where *x*_*j*_^min^ is the lower limit of the *j*^th^ optimization parameter, and *x*_*j*_^max^ is the upper limit of the *j*^th^ optimization parameter.

Apart from the initialization phase, the algorithm is divided into three phases which are employed bee phase, onlooker bee phase, and scout bee phase. All three phases are repeated until the termination criterion is satisfied or until the maximum number of fitness evaluations (*MFE*) is reached.

In the employed bee phase, the neighboring food source ${\bar{v}}^{t}{}_{i}$ of the current food source ${\bar{x}}^{t}{}_{i}$ is discovered by employed bees as proposed in [16], as shown in Eq (7).

$$v_{i,j}^{t}=x_{i,j}^{t}+rand\left[-1,1\right]\cdot \left(x_{i,j}^{t}-x_{k,j}^{t}\right)$$ (7)

Where *v*^*t*^_*i*,*j*_ is the *j*^th^ optimization parameter of ${\bar{v}}^{t}{}_{i}$. An integer *j* is randomly selected in the interval of [1, *D*]. The index of food source *k* is randomly selectedin the interval of [1, *SN*], and *k* must not be equal to *i* to prevent the value of *v*^*t*^_*i*,*j*_ from being the same as that of *x*^*t*^_*i*,*j*_.

If the new food source ${\bar{v}}^{t}{}_{i}$ provides a better fitness value than the current food source ${\bar{x}}^{t}{}_{i}$, i.e., $Fit({\bar{v}}^{t}{}_{i})$ > $Fit({\bar{x}}^{t}{}_{i})$, the employed bee forgets the current food source and memorizes the new one.

The reinforcement vector ${\bar{r}}^{t}$ for the next iteration is then updated by using the linear reward-penalty scheme or Bush-Mosteller scheme [17–18] in Eqs (8) and (9). If the new food source provides a better fitness value, the employed bee not only replaces the current food source with the new food source, but also gives a larger reinforcement value as a reward to the selected dimension while the reinforcement values of other dimensions are smaller as shown in Eq (8). On the other hand, if the new food source does not provide a better fitness value, the new food source is ignored and a penalty is given to the selected dimension by decreasing the reinforcement value while the reinforcement values of other dimensions are increased as shown in Eq (9).

If $Fit({\bar{v}}^{t}{}_{i})$ > $Fit({\bar{x}}^{t}{}_{i})$ then

$$r_{j}^{t+1}=\left\{ \begin{array}{l} r_{j}^{t}+\alpha \left(1-r_{j}^{t}\right),j=d\\ r_{j}^{t}\times \left(1-\alpha \right),j\neq d \end{array}\right.$$ (8)

If $Fit({\bar{v}}^{t}{}_{i})$ < = $Fit({\bar{x}}^{t}{}_{i})$ then

$$r_{j}^{t+1}=\left\{ \begin{array}{l} r_{j}^{t}\times \left(1-\beta \right),j=d\\ \frac{\beta }{D-1}+r_{j}^{t}\times \left(1-\beta \right),j\neq d \end{array}\right.$$ (9)

Where *j* and *d* ∈ {1, 2, 3, …, *D*} and *d* is the randomly dimension selected by an employed bee. *α* and *β* are the degree of reward and the degree of penalty, respectively. The reinforcement vector is updated every time an employed bee finds a candidate food source. Therefore, if *EB* is the number of employed bees, the reinforcement vector is updated *EB* times per iteration. After updating, the sum of the reinforcement values is always equal to 1. The proof is shown in S1 Appendix. The values of *α* and *β* are shown in Eq (10).

$$\alpha =\beta =\frac{Fit\left({\vec{x}}_{i}^{t}\right)}{\sum_{n=1}^{SN}Fit\left({\vec{x}}_{n}^{t}\right)}$$ (10)

In the onlooker bee phase, each onlooker bee selects a candidate food source provided by employed bee depending on a probability associated with the fitness values as in Eq (3). In contrast to the original ABC algorithm, an onlooker bee discovers new food source using Eq (11) and replaces the current food source with the new food source if the new food source is more profitable.

$$v_{i,j}^{t}=x_{i,k}^{t}+\Phi_{i,j}^{t}\cdot r_{j}^{t}\cdot \left(x_{i,k}^{t}-x_{BSF,k}^{t}\right)$$ (11)

Where *v*^*t*^_*i*,*j*_ is the optimization parameter of a neighboring food source ${\bar{v}}^{t}{}_{i}$ at the dimension *j* ∈ {1, 2, 3, …, *D*} in the iteration *t*. *k* ∈ {1, 2, 3, …, *D*} is a random number indicating a random dimension for all *j*. Φ ∈ [–1, 1] is a random real number. *x*^*t*^_*BSF*,*k*_ is the optimization parameter of the global best food source found so far at a random dimension *k*.

The new search equation in Eq (11) is designed to enhance the exploitation. Rather than updating the value in a dimension, an onlooker bee exploits around the current food source by updating all dimensions with different weights. The reinforcement vector (*r*_*j*_^*t*^) indicates how much an onlooker bee focuses on updating each dimension. If the value in a dimension of the reinforcement vector is equal to 1, and all other dimensions are equal to 0, an onlooker bee will update only one dimension as in the original ABC algorithm which is one-dimension update process as aforementioned. Conversely, if the values in all dimensions of the reinforcement vector are similar, an onlooker bee updates all dimensions with similar weights. In this case, the onlooker bee may not be able to find a better food source.

**Fig 2:**
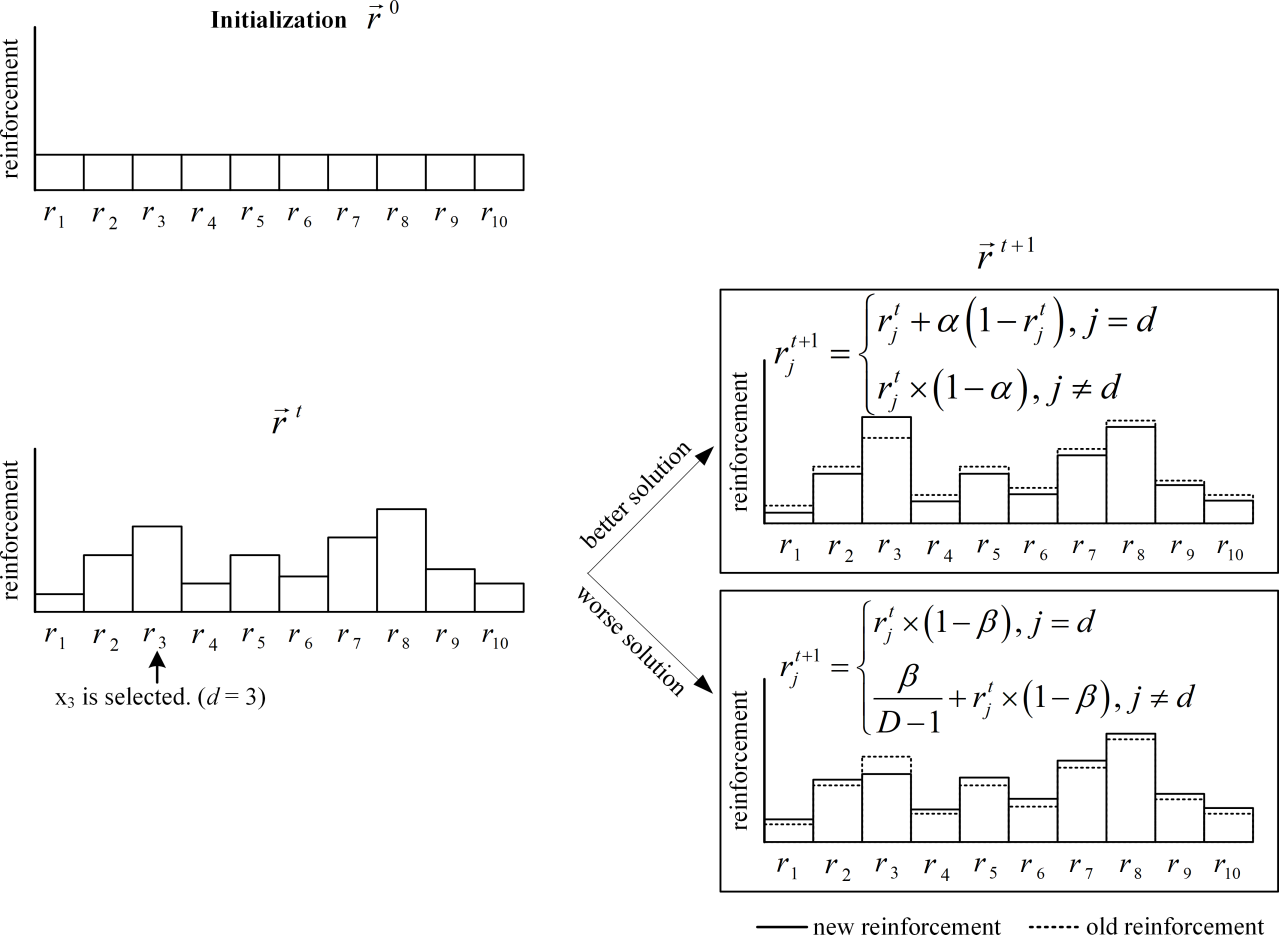
An example of the reinforcement vector update.

The degree of reward and penalty control how much the reinforcement values are adapted. They have adjusted adaptively according to the fitness value of a candidate food source found by an employed bee. According to the win-stay lose-shift strategy in the animals’ foraging behavior [15], if animals find food in an area, they will keep finding more food in that area. Otherwise, they will move to another area. In the proposed algorithm, if an employed bee can find a better food source in dimension *d* of the candidate food source, the onlooker bees will assign more weight to dimension *d* by Eq (8). On the other hand, if an employed bee cannot find a better food source, the smaller weight value is assigned to the dimension *d*, by Eq (9). If the candidate food source is better than the current food source, the value of the reinforcement value in the dimension *d* increases. Otherwise, the reinforcement value will be decreased. In some cases, the candidate food source can have a lower value of reinforcement vector (*r*_*j*_^*t*^) than the current food source but its fitness value is higher than those of other food sources. This means that the candidate food source is a local optimum food source and the negative reinforcement value is assigned. This process allows the onlooker bees to escape from a local optimum food source by updating the values in other dimensions with higher weights. However, if there is any optimum value in a dimension (*j*) that is far away from others, the explorative behavior of employed bees, Eq (7), is required to handle the case. Fig 2 shows an example of the reinforcement vector updating. If an employed bee updates the value in dimension 4, and the fitness value of the candidate food source is better, the value of *r*_*4*_^*t+1*^ increases. Otherwise, the value of *r*_*4*_^*t+1*^ decreases.

In the scout bee phase, if the number of unsuccessful attempts to find a better neighbor for any food source exceeds the limit, the food source is abandoned and a scout bee randomly discovers a new food source as in [16].

The pseudo-code of the R-ABC algorithm is presented in Algorithm 1.

**Algorithm 1. The R-ABC algorithm.**

**Initialization:**

1: Generate a set of random food sources;

2: Assign employed bees to the food sources;

3: Generate initial range *R*;

4: Evaluate the food sources; *FEs* = Number of food sources;

5: Find the best food source;

6: WHILE termination criterion is not satisfied

**Employed Bee Phase:**

7:        FOR each food source DO

8:            Update position of employed bee using Eq (7);

9:            Evaluate the new position; *FEs* = *FEs*+1;

10:            IF fitness value of new position is better

11:                Reset *TRIAL*;

12:                Replace the current food source with the new one;

13:                Apply positive reinforcement using Eq (8);

14:            ELSE

15:                *TRIAL* = *TRIAL*+1;

16:                Apply negative reinforcement using Eq (9);

17:            END IF

18:        END FOR

19:        Calculate probability for selecting food source in the onlooker bee phase;

**Onlooker Bee Phase:**

20:        FOR each onlooker bee DO

21:            Select a food source based on probability;

22:            Update position of onlooker bee using Eq (11);

23:            Evaluate the new position; *FEs* = *FEs*+1;

24:            IF fitness value of new position is better

25:                Reset *TRIAL*;

26:                Replace the current food source with the new one;

27:            ELSE

28:                *TRIAL* = *TRIAL*+1;

29:            END IF

30:        END FOR

31:        Find the best food source;

**Scout Bee Phase:**

32:        FOR each food source DO

33:            IF *TRIAL* > *LIMIT*

34:                Abandon the food source and send a scout bee to discovery a new food source;

35:                Evaluate the new position; *FEs* = *FEs*+1;

36:            END IF

37:        END FOR

38: END WHILE

The effect of the reinforcement learning integrated into the proposed algorithm is to adjust the range of the area searched in the onlooker bee phase. There are two differences between the original ABC and the R-ABC algorithms.

The first difference relates to the information shared between employed bees and onlooker bees. In both algorithms, an employed bee updates one dimension at a time to find a new food source and then shares some information with onlooker bees. In the original ABC algorithm, the information which employed bees share with an onlooker bee is only the quality of the food source in terms of the fitness values. Then each onlooker bee uses the fitness values of food sources to select a candidate food source in Eq (7). In the R-ABC algorithm, each employed bee shares not only the quality of food source but also which dimension should be changed to find a better food source. This additional information is shared in the terms of the reinforcement. If an employed bee changes a dimension and finds a better food source, it gives a positive reinforcement to the dimension. On the other hand, if it changes a dimension and finds a worse food source, it gives a negative reinforcement to the dimension. Each onlooker bee uses the reinforcement to update its position as shown in Eq (11).

The second difference between the original ABC and the R-ABC algorithms is the number of dimensions updated by an onlooker bee. In the original ABC algorithm, only one dimension is changed by an onlooker bee. In the R-ABC algorithm, all dimensions are changed by an onlooker bee with different ranges according to the reinforcement. A dimension which is changed and gives a better food source has a wider update range. On the other hand, a dimension which is changed and produces a worse food source has a narrower update range.

For example, suppose bees fly along the x-axis, y-axis, and z-axis to find the best food source, in other words, a 3*D* problem. An employed bee explores a new food source by changing only one dimension at a time, i.e., it moves along only one axis. If an employed bee flies along the y-axis and finds a better food source, it tells onlooker bees that they should move along the y-axis too. Each onlooker bee moves in all three dimensions, but it tends to move along the y-axis more than other dimensions. On the other hand, if an employed bee flies along the y-axis and finds a worse food source, it tells onlooker bees that they should not move very much along this dimension.

The reinforcement varies over time depending on which dimension is selected and the quality of the food sources found by employed bees. For example, if the first employed bee finds a better food source by changing dimension *d*, it gives a positive reinforcement to dimension *d*, i.e., the reinforcement of dimension *d* increases while those of other dimensions decrease. The higher the quality of the new food source is, the more the positive reinforcement is given to dimension *d*. Later, if the second employed bee finds a better food source by changing another dimension, it gives a positive reinforcement to that dimension and the reinforcement of other dimensions including *d* decrease.

Although in both the R-ABC and the BSF-ABC algorithms, the values of all dimensions are updated. In the BSF-ABC algorithm, the values in all dimensions of each food source are updated according to the best-so-far food source as the multiplier of the error correction for this solution update [9]. In the R-ABC algorithm, the values of all dimensions are modified with different magnitudes. The magnitude of each dimension varies according to the fitness values of new food sources found by employed bees.

**Fig 3:**
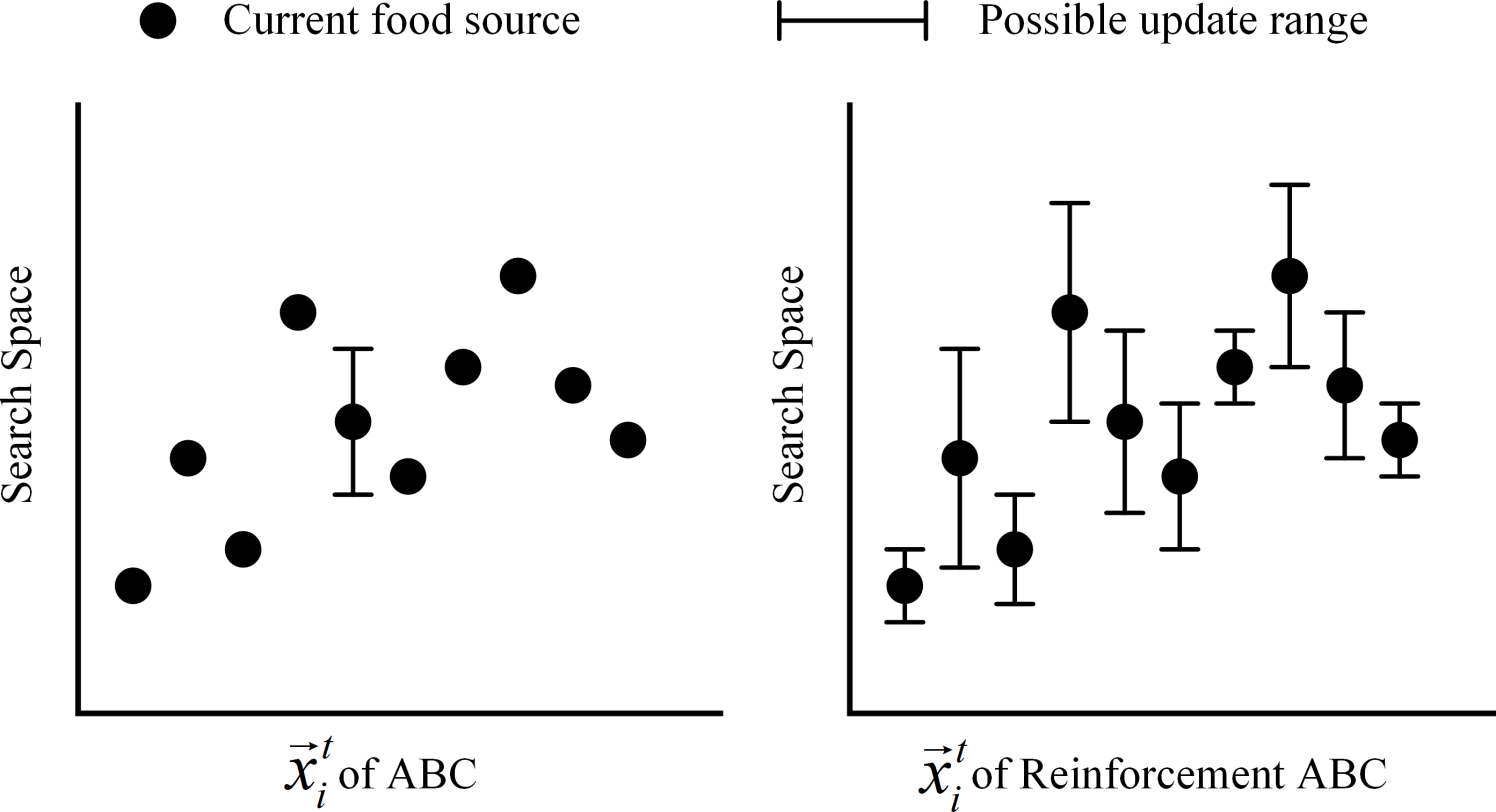
An example of changing 10*D* reinforcement.

Fig 3 shows an example of a 10*D* reinforcement vector changing over time. At the initialization, the reinforcement for all dimensions is equal. Each reinforcement is changed when an employed bee discovers a new food source. At time *t*, the employed bee finds a new food source by changing dimension *x*_3_ and evaluates the new food source. At time *t*+1, the values of reinforcement are changed according to the quality of the new food source.

**Fig 4:**
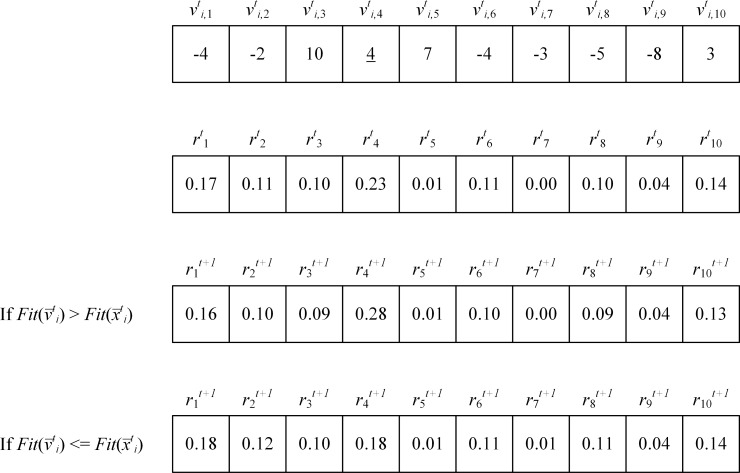
Possible update ranges of food sources.

The difference of the position update between the original ABC algorithm and the R-ABC algorithm is shown in Fig 4. An onlooker bee in the original ABC algorithm selects a random dimension to update the position of food source, but an onlooker of the R-ABC algorithm updates all dimensions within different ranges according to the positive-negative reinforcement associated with the food source discovery in the employed bee phase. The dimension that provides a better fitness value when changed has the widest possible update range.

## Experimental settings

To evaluate the performance of the R-ABC algorithm, we tested the proposed technique compared with the state-of-the-art algorithms on widely-used numerical benchmark functions. The R-ABC algorithm was evaluated by applying it to a set of numerical benchmark functions focusing on two characteristics as follows.

- Unimodal and multi-modal: Modality of a function corresponds to the number of peaks in the function surface [19]. Finding the global optimum of a multi-modal function is more difficult than that of a unimodal function because algorithms may become trapped at one of the peaks which is a local optimum solution.
- Separable and non-separable: Separability of a function is a measure of the whether each parameter in a solution is independent to the other parameters [19]. Dealing with a separable benchmark function is easier than dealing with a non-separable one because a separable function can be decomposed into sub-functions and they can be optimized separately [20].

Thus, we used four categories of benchmark functions:- unimodal separable functions (US), unimodal non-separable functions (UN), multimodal separable functions (MS), and multimodal non-separable functions (MN). We chose two benchmark functions from each category. The description of the selected benchmark functions [19] is shown in Table 1. In addition, to evaluate the performance of the R-ABC algorithm on functions with more difficulty, we employed seven shifted functions from CEC2005 [21] and six hybrid functions from CEC2014 [22] to test. The CEC2005’s shifted functions and the CEC2014’s hybrid functions are shown in Table 2 and Table 3, respectively. The objective is to find the minimum output values from the functions.

**Table 1:** Numerical benchmark functions.

Function name (Type)	Function	Range	Global minimum *f*(x*) = 0
Sphere (US)	$f_{1}\left(\vec{x}\right)=\sum_{i=1}^{D}x_{i}^{2}$	[–100, 100]	*f*_1_(0,…,0)
Sum Squares (US)	$f_{2}\left(\vec{x}\right)=\sum_{i=1}^{D}ix_{i}^{2}$	[–10, 10]	*f*_2_(0,…,0)
Dixon-Price (UN)	$f_{3}\left(\vec{x}\right)={\left(x_{1}-1\right)}^{2}+{\sum_{i=2}^{D}i\left(2x_{i}^{2}-x_{i-1}\right)}^{2}$	[–10, 10]	$f_{3}\left(2^{-\left(\frac{2^{i}-2}{2^{i}}\right)}\right)$
Rosenbrock (UN)	$f_{4}\left(\vec{x}\right)=\sum_{i=1}^{D-1}\left(100{\left(x_{i+1}-x_{i}^{2}\right)}^{2}+{\left(x_{i}-1\right)}^{2}\right)$	[–30, 30]	*f*_4_(1,…,1)
Rastrigin (MS)	$f_{5}\left(\vec{x}\right)=10D+\sum_{i=1}^{D}\left(x_{i}^{2}-10\cos\left(2\pi x_{i}\right)\right)$	[-5.12, 5.12]	*f*_5_(0,…,0)
Schwefel (MS)	$f_{6}\left(\vec{x}\right)=418.9829D-\sum_{i=1}^{D}x_{i}\sin\left(\sqrt{\left|x_{i}\right|}\right)$	[–500, 500]	*f*_6_(420.9687,…,420.9687)
Ackley (MN)	$f_{7}\left(\vec{x}\right)=20+e-20e^{\left(-0.2\sqrt{\frac{1}{D}\sum_{i=1}^{D}x_{i}^{2}}\right)}-e^{\left(\frac{1}{D}\sum_{i=1}^{D}\cos\left(2\pi x_{i}\right)\right)}$	[–32, 32]	*f*_7_(0,…,0)
Griewank (MN)	$f_{8}\left(\vec{x}\right)=\left(\sum_{i=1}^{D}\frac{x_{i}^{2}}{4000}\right)-\left(\prod_{i=1}^{D}\cos\left(\frac{x_{i}}{\sqrt{i}}\right)\right)+1$	[–600, 600]	*f*_8_(0,…,0)

**Table 2:** CEC2005’s shifted functions.

Function Name	Function
Shifted Function 1	*F*_6_: Shifted Rosenbrock’s Function
Shifted Function 2	*F*_7_: Shifted Rotated Griewank’s Function without Bounds
Shifted Function 3	*F*_8_: Shifted Rotated Ackley’s Function with Global Optimum on Bounds
Shifted Function 4	*F*_9_: Shifted Rastrigin’s Function
Shifted Function 5	*F*_10_: Shifted Rotated Rastrigin’s Function
Shifted Function 6	*F*_11_: Shifted Rotated Weierstras Function
Shifted Function 7	*F*_12_: Schwefel’s Problem 2.13

**Table 3:** CEC2014’s hybrid functions.

Function name	Subcomponent *g*_i_(*x*)	Percentage of *g*_i_(*x*)
Hybrid Function 1	*g*_1_: Modified Schwefel's Function	0.3
	*g*_2_: Rastrigin’s Function	0.3
	*g*_3_: High Conditioned Elliptic Function	0.4
Hybrid Function 2	*g*_1_: Bent Cigar Function	0.3
	*g*_2_: HGBat Function	0.3
	*g*_3_: Rastrigin’s Function	0.4
Hybrid Function 3	*g*_1_: Griewank’s Function	0.2
	*g*_2_: Weierstrass Function	0.2
	*g*_3_: Rosenbrock’s Function	0.3
	*g*_4_: Scaffer’s F6 Function	0.3
Hybrid Function 4	*g*_1_: HGBat Function	0.2
	*g*_2_: Discus Function	0.2
	*g*_3_: Expanded Griewank’s plus Rosenbrock’s Function	0.3
	*g*_4_: Rastrigin’s Function	0.3
Hybrid Function 5	*g*_1_: Scaffer’s F6 Function	0.1
	*g*_2_: HGBat Function	0.2
	*g*_3_: Rosenbrock’s Function	0.2
	*g*_4_: Modified Schwefel’s Function	0.2
	*g*_5_: High Conditioned Elliptic Function	0.3
Hybrid Function 6	*g*_1_: Katsuura Function	0.1
	*g*_2_: HappyCat Function	0.2
	*g*_3_: Expanded Griewank’s plus Rosenbrock’s Function	0.2
	*g*_4_: Modified Schwefel’s Function	0.2
	*g*_5_: Ackley’s Function	0.3

The R-ABC algorithm was compared with seven algorithms listed below.

- Adaptive Population ABC (APABC) [5]
- Adaptive and Hybrid ABC (aABC) [11]
- Best-So-Far ABC (BSF-ABC) [9]
- ABC with a variable search strategy (ABCVSS) [4]
- ABC [16]
- Starling Particle Swarm Optimization (Starling PSO) [23]
- Adaptive Differential Evolution with Optional External Archive (JADE) [24]

Code for BSF-ABC, ABC, and Starling PSO was provided by the original authors, whereas code for APABC, aABC, ABCVSS, and JADE was rewritten based on published reports and validated with the results of the original research, except for APABC because the published report does not provide the final results. As we are focusing on the issue of very high-dimensional problems, the experiments were conducted to solve 100*D*, 500*D*, 700*D*, and 900*D* problems, except for the CEC2014’s hybrid functions which are defined for 100*D*. The maximum number of fitness evaluations (*MFE*) is set to 30000. If the number of function evaluations reaches *MFE* or the function value reaches the global optimum, the run is terminated. However, the BSF-ABC requires a pre-defined maximum number of iterations (*MCN*) because it uses the values of the current iteration number and *MCN* to update scout bees’ positions. Therefore, the comparison with BSF-ABC was conducted separately based on *MCN* = 10000. Moreover, to validate the effectiveness of the reinforcement vector, we also conducted the experiments with random vectors. The value of each element in a random vector is uniformly random, and the sum of the random values is equal to 1. Each experiment was run 20 times on 2.00 GHz Intel® Xeon® CPU with 4.00 GB memory, and all memory was cleared before each run. The summary of the control parameters is shown in Table 4. The values of adaptivity coefficients *α* and crossover rate *γ* providing the best results for each function in [11] were selected to be used in aABC algorithm as shown in Table 5, while the values of adaptivity coefficients *α* and crossover rate *γ* used in the CEC2005’s shifted functions and the CEC2014’s hybrid functions were 0.5 and 0.5, respectively.

**Table 4:** Values of control parameters.

Parameter	Symbol	R-ABC	APABC	BSF-ABC	aABC	ABCVSS	ABC	Starling PSO	JADE
Population size	*N*	50	-	50	50	50	50	50	50
Maximum number of food sources	*SN*_max_	-	35	-	-	-	-	-	-
Minimum number of food sources	*SN*_min_	-	20	-	-	-	-	-	-
Trial limit	*LIMIT*	100	-	100	100	100	100	-	-
Statistical period	*T*	-	20	-	-	-	-	-	-
Maximum percentage of scout bees’ position adjustment	*ω*_max_	-	-	0.2	-	-	-	-	-
Minimum percentage of scout bees’ position adjustment	*ω*_min_	-	-	0.2	-	-	-	-	-
Maximum inertia weight	*w*_max_	-	-	-	-	-	-	0.4	-
Minimum inertia weight	*w*_min_	-	-	-	-	-	-	0.2	-
Weight of personal best	*c*_1_	-	-	-	-	-	-	2	-
Weight of global best	*c*_2_	-	-	-	-	-	-	2	-
Parameter adaptation rate	*c*	-	-	-	-	-	-	-	0.1
Greediness of mutation strategy	*p*	-	-	-	-	-	-	-	0.05

**Table 5:** Values of adaptivity coefficients and crossover rates used in aABC algorithm.

Function	Adaptivity coefficient	Crossover rate
Unimodal Separable		
Sphere	0.5	0.5
Sum Squares	0.5	0.5
Unimodal Non-separable		
Dixon-Price	0.25	1
Rosenbrock	0.5	0.7
Multimodal Separable		
Rastrigin	0.5	0.8
Schwefel	0.25	0.7
Multimodal Non-separable		
Ackley	0	0.5
Griewank	0.5	0.5

## Results and discussion

### Basic numerical benchmark functions

The results of the algorithm for basic benchmark functions are presented in Tables 6–8. Tables 6 and 8 show the average final values of the R-ABC and the compared algorithms. However, the R-ABC and the BSF-ABC algorithms may not consume the same number of fitness evaluations to provide the results in Table 8. The “*D*” column is the number of dimensions. The “Mean” row is the arithmetic mean of output values. The “Min” and “Max” rows present the minimum and maximum outputs respectively. The “SD” row is the standard deviation of the results. For “Mean”, “Min”, “Max”, and “SD” rows, the values which are smaller than 1E-308 are considered as 0. The best results are marked with boldface. Note that the global minimum value of Ackley function is computationally 8.88E-16, but considered as 0. The “Sig” row is the comparison results from the Wilcoxon’s rank sum test to show whether the final results from all runs between the R-ABC and each competitor are significantly different. The final results which are smaller than 1E-308 are replaced by 0 before the test. The “+”, “=“, and “-” symbols mean that the final results of the R-ABC algorithm are better than, similar to, and worse than those of a competitor at 0.05 significant level, respectively. Although the results of the R-ABC and BSF-ABC algorithms for the Schwefel function are the same in Table 8, the actual values are different but very trivial. Table 7 shows the results of algorithm ranking by the Friedman’s test by using KEEL [25–26].

**Table 6:** Results for basic benchmark functions (*MFE* = 30000).

*D*	Stat	R-ABC	APABC	aABC	ABCVSS	ABC	Starling PSO	JADE
		Sphere						
100	Mean	**4.31E-13**	1.53E+03	5.48E+00	2.30E+05	1.00E+02	5.92E+03	6.69E-04
	Min	**1.26E-17**	7.87E+02	9.57E-01	1.82E+05	3.51E+00	6.60E+01	1.01E-04
	Max	**6.16E-12**	3.25E+03	1.69E+01	2.58E+05	8.05E+02	1.05E+04	1.97E-03
	SD	**1.41E-12**	5.77E+02	4.10E+00	2.11E+04	1.76E+02	4.91E+03	4.59E-04
	Sig		+	+	+	+	+	+
500	Mean	**6.17E-03**	2.87E+05	3.98E+05	1.47E+06	6.20E+05	4.85E+05	1.75E+04
	Min	**7.69E-11**	2.18E+05	3.47E+05	1.36E+06	5.65E+05	2.78E+05	1.46E+04
	Max	**5.41E-02**	3.89E+05	4.41E+05	1.55E+06	6.75E+05	5.71E+05	2.52E+04
	SD	**1.31E-02**	4.66E+04	2.53E+04	3.61E+04	3.03E+04	6.86E+04	2.40E+03
	Sig		+	+	+	+	+	+
700	Mean	**7.79E-02**	6.47E+05	8.84E+05	2.11E+06	1.20E+06	8.18E+05	5.64E+04
	Min	**2.26E-05**	4.81E+05	8.41E+05	2.04E+06	1.07E+06	4.58E+05	4.48E+04
	Max	**7.81E-01**	8.00E+05	9.19E+05	2.18E+06	1.35E+06	1.01E+06	6.62E+04
	SD	**1.83E-01**	8.67E+04	2.25E+04	3.70E+04	7.31E+04	1.53E+05	6.82E+03
	Sig		+	+	+	+	+	+
900	Mean	**7.04E-02**	1.03E+06	1.44E+06	2.76E+06	1.82E+06	1.21E+06	1.05E+05
	Min	**2.18E-05**	8.55E+05	1.36E+06	2.68E+06	1.68E+06	6.05E+05	8.44E+04
	Max	**5.04E-01**	1.22E+06	1.49E+06	2.81E+06	1.98E+06	1.47E+06	1.27E+05
	SD	**1.22E-01**	9.94E+04	3.13E+04	3.69E+04	9.14E+04	2.54E+05	1.17E+04
	Sig		+	+	+	+	+	+
		**Sum Squares**						
100	Mean	**1.73E-12**	7.01E+02	2.07E+00	1.09E+05	3.97E+01	4.83E+03	4.39E-04
	Min	**2.43E-18**	3.75E+02	1.89E-01	7.04E+04	1.42E+00	1.62E+02	7.41E-05
	Max	**3.28E-11**	1.14E+03	8.76E+00	1.27E+05	4.10E+02	1.48E+04	1.05E-03
	SD	**7.32E-12**	2.07E+02	2.19E+00	1.31E+04	1.01E+02	3.26E+03	3.34E-04
	Sig		+	+	+	+	+	+
500	Mean	**2.58E-02**	7.20E+05	9.59E+05	3.59E+06	1.55E+06	1.06E+06	3.76E+04
	Min	**2.01E-06**	5.39E+05	8.57E+05	3.30E+06	1.33E+06	5.57E+05	2.69E+04
	Max	**2.00E-01**	9.90E+05	1.08E+06	3.80E+06	1.72E+06	1.34E+06	5.02E+04
	SD	**5.84E-02**	1.21E+05	5.56E+04	1.17E+05	1.16E+05	1.98E+05	6.06E+03
	Sig		+	+	+	+	+	+
700	Mean	**5.84E-02**	2.29E+06	3.00E+06	7.32E+06	4.06E+06	2.63E+06	1.66E+05
	Min	**3.19E-07**	1.89E+06	2.83E+06	7.03E+06	3.68E+06	1.58E+06	1.13E+05
	Max	**3.39E-01**	2.76E+06	3.15E+06	7.63E+06	4.43E+06	3.20E+06	2.12E+05
	SD	**9.19E-02**	2.66E+05	1.02E+05	1.60E+05	2.31E+05	4.13E+05	2.36E+04
	Sig		+	+	+	+	+	+
900	Mean	**2.97E-01**	4.64E+06	6.38E+06	1.21E+07	8.18E+06	4.79E+06	3.98E+05
	Min	**2.23E-04**	4.01E+06	6.01E+06	1.11E+07	7.46E+06	2.97E+06	3.40E+05
	Max	**2.15E+00**	5.37E+06	6.70E+06	1.26E+07	8.93E+06	6.43E+06	4.86E+05
	SD	**5.21E-01**	4.51E+05	1.84E+05	3.28E+05	4.13E+05	9.11E+05	3.78E+04
	Sig		+	+	+	+	+	+
		**Dixon-Price**						
100	Mean	**2.83E-01**	2.94E+02	5.98E+02	1.91E+07	1.83E+03	2.87E+05	2.73E+01
	Min	**1.85E-01**	6.30E+01	6.51E+01	2.04E+06	7.60E+01	6.86E+02	7.67E+00
	Max	**5.65E-01**	4.00E+03	1.46E+03	2.65E+07	2.76E+04	1.18E+06	4.18E+01
	SD	**8.26E-02**	8.72E+02	4.76E+02	5.56E+06	6.09E+03	3.56E+05	9.04E+00
	Sig		+	+	+	+	+	+
500	Mean	**4.38E+00**	6.70E+07	2.81E+08	8.15E+08	2.74E+08	2.13E+08	5.07E+05
	Min	**3.10E-01**	3.10E+07	2.29E+08	7.53E+08	2.25E+08	1.29E+08	2.78E+05
	Max	**1.78E+01**	1.64E+08	3.53E+08	8.64E+08	3.51E+08	3.21E+08	7.13E+05
	SD	**4.74E+00**	2.67E+07	3.64E+07	3.07E+07	3.05E+07	5.00E+07	1.07E+05
	Sig		+	+	+	+	+	+
700	Mean	**1.59E+01**	3.21E+08	8.10E+08	1.65E+09	7.92E+08	5.07E+08	3.06E+06
	Min	**4.70E-01**	2.62E+08	7.12E+08	1.51E+09	6.92E+08	3.61E+08	2.21E+06
	Max	**1.06E+02**	4.36E+08	8.86E+08	1.78E+09	8.80E+08	6.95E+08	3.98E+06
	SD	**2.81E+01**	4.15E+07	5.26E+07	7.28E+07	5.55E+07	9.53E+07	5.16E+05
	Sig		+	+	+	+	+	+
900	Mean	**3.14E+01**	8.32E+08	1.66E+09	2.82E+09	1.64E+09	1.08E+09	8.77E+06
	Min	**8.91E-01**	6.95E+08	1.42E+09	2.49E+09	1.46E+09	5.53E+08	6.53E+06
	Max	**3.26E+02**	1.28E+09	1.90E+09	2.92E+09	1.79E+09	1.41E+09	1.20E+07
	SD	**7.51E+01**	1.20E+08	1.29E+08	1.12E+08	8.15E+07	2.21E+08	1.40E+06
	Sig		+	+	+	+	+	+
		**Rosenbrock**						
100	Mean	**4.23E+00**	1.79E+04	4.81E+03	8.95E+08	1.28E+04	7.01E+04	3.09E+02
	Min	**5.22E-05**	7.90E+02	1.30E+03	4.84E+08	1.05E+03	9.31E+03	2.07E+02
	Max	**1.68E+01**	3.38E+05	2.19E+04	1.09E+09	1.13E+05	2.77E+05	4.26E+02
	SD	**5.59E+00**	7.54E+04	4.70E+03	1.54E+08	2.47E+04	7.13E+04	7.30E+01
	Sig		+	+	+	+	+	+
500	Mean	**1.63E+02**	4.14E+08	1.47E+09	6.69E+09	2.23E+09	1.73E+09	5.17E+06
	Min	**8.33E-01**	2.90E+08	1.22E+09	5.33E+09	1.80E+09	4.10E+08	3.39E+06
	Max	**4.95E+02**	1.04E+09	1.82E+09	7.18E+09	2.64E+09	2.63E+09	6.87E+06
	SD	**1.68E+02**	1.61E+08	1.47E+08	4.09E+08	1.97E+08	5.16E+08	9.52E+05
	Sig		+	+	+	+	+	+
700	Mean	**2.07E+02**	1.39E+09	3.56E+09	9.69E+09	4.67E+09	3.28E+09	1.88E+07
	Min	**4.95E+00**	9.14E+08	3.14E+09	9.22E+09	4.07E+09	1.78E+09	1.10E+07
	Max	**7.08E+02**	1.77E+09	3.90E+09	1.03E+10	5.37E+09	4.84E+09	2.88E+07
	SD	**2.30E+02**	2.37E+08	2.07E+08	2.97E+08	3.81E+08	7.28E+08	4.00E+06
	Sig		+	+	+	+	+	+
900	Mean	**2.61E+02**	2.83E+09	6.12E+09	1.28E+10	7.43E+09	5.30E+09	4.43E+07
	Min	**8.39E-02**	2.17E+09	5.60E+09	1.22E+10	6.53E+09	3.10E+09	3.36E+07
	Max	**1.07E+03**	3.95E+09	6.56E+09	1.35E+10	8.16E+09	7.40E+09	6.73E+07
	SD	**3.28E+02**	3.79E+08	2.48E+08	3.26E+08	4.16E+08	1.24E+09	8.54E+06
	Sig		+	+	+	+	+	+
		**Rastrigin**						
100	Mean	**8.01E-09**	2.24E+01	1.64E+02	1.30E+03	1.60E+02	4.18E+02	2.97E+02
	Min	**0.00E+00**	9.17E+00	1.41E+02	7.36E+02	1.08E+02	2.06E+02	2.65E+02
	Max	**1.48E-07**	1.71E+02	1.87E+02	1.50E+03	1.96E+02	6.22E+02	3.24E+02
	SD	**3.30E-08**	3.50E+01	1.34E+01	2.01E+02	2.25E+01	1.06E+02	1.62E+01
	Sig		+	+	+	+	+	+
500	Mean	**1.58E-01**	3.15E+03	4.55E+03	8.42E+03	4.68E+03	4.32E+03	3.88E+03
	Min	**1.22E-09**	2.84E+03	4.27E+03	7.32E+03	4.12E+03	3.80E+03	3.73E+03
	Max	**8.14E-01**	3.92E+03	4.77E+03	8.79E+03	5.10E+03	5.02E+03	4.09E+03
	SD	**2.12E-01**	2.22E+02	1.50E+02	2.99E+02	2.24E+02	3.73E+02	9.43E+01
	Sig		+	+	+	+	+	+
700	Mean	**2.87E+00**	5.74E+03	7.77E+03	1.20E+04	7.93E+03	6.44E+03	5.83E+03
	Min	**4.06E-04**	5.12E+03	7.33E+03	1.10E+04	7.43E+03	4.32E+03	5.59E+03
	Max	**1.91E+01**	6.13E+03	7.93E+03	1.24E+04	8.47E+03	7.46E+03	6.01E+03
	SD	**4.91E+00**	2.68E+02	1.80E+02	3.12E+02	2.85E+02	8.14E+02	9.77E+01
	Sig		+	+	+	+	+	+
900	Mean	**3.36E+00**	8.79E+03	1.10E+04	1.57E+04	1.13E+04	9.15E+03	7.92E+03
	Min	**2.50E-02**	7.83E+03	1.05E+04	1.55E+04	1.09E+04	6.53E+03	7.62E+03
	Max	**1.14E+01**	9.57E+03	1.13E+04	1.59E+04	1.18E+04	1.06E+04	8.10E+03
	SD	**3.70E+00**	4.25E+02	2.22E+02	1.21E+02	2.39E+02	9.96E+02	1.22E+02
	Sig		+	+	+	+	+	+
		**Schwefel**						
100	Mean	**1.29E-03**	7.96E+02	1.24E+04	3.24E+04	9.92E+03	1.53E+04	1.57E+04
	Min	**1.27E-03**	2.38E+02	1.13E+04	2.68E+04	7.97E+03	1.31E+04	1.43E+04
	Max	**1.45E-03**	5.21E+03	1.36E+04	3.55E+04	1.08E+04	1.84E+04	1.66E+04
	SD	**4.01E-05**	1.05E+03	6.57E+02	2.33E+03	7.39E+02	1.31E+03	5.89E+02
	Sig		+	+	+	+	+	+
500	Mean	**8.98E-01**	9.58E+04	1.30E+05	1.95E+05	1.25E+05	1.30E+05	1.51E+05
	Min	**7.23E-03**	9.08E+04	1.20E+05	1.85E+05	1.18E+05	1.14E+05	1.47E+05
	Max	**3.94E+00**	1.07E+05	1.38E+05	2.00E+05	1.34E+05	1.43E+05	1.54E+05
	SD	**1.07E+00**	3.77E+03	4.82E+03	3.39E+03	4.92E+03	7.84E+03	2.09E+03
	Sig		+	+	+	+	+	+
700	Mean	**3.77E+01**	1.61E+05	2.01E+05	2.75E+05	2.00E+05	1.94E+05	2.25E+05
	Min	**1.05E-02**	1.47E+05	1.90E+05	2.62E+05	1.86E+05	1.84E+05	2.19E+05
	Max	**3.41E+02**	1.73E+05	2.12E+05	2.80E+05	2.08E+05	2.12E+05	2.29E+05
	SD	**7.64E+01**	5.52E+03	5.74E+03	3.98E+03	6.66E+03	9.06E+03	2.81E+03
	Sig		+	+	+	+	+	+
900	Mean	**1.46E+02**	2.32E+05	2.79E+05	3.58E+05	2.77E+05	2.61E+05	3.01E+05
	Min	**1.37E-02**	2.24E+05	2.64E+05	3.50E+05	2.63E+05	2.51E+05	2.96E+05
	Max	**1.79E+03**	2.43E+05	2.87E+05	3.64E+05	2.86E+05	2.79E+05	3.04E+05
	SD	**3.96E+02**	5.87E+03	5.70E+03	3.45E+03	6.08E+03	7.52E+03	2.22E+03
	Sig		+	+	+	+	+	+
		**Ackley**						
100	Mean	**2.44E-07**	7.95E-01	3.35E+00	2.05E+01	9.06E+00	5.71E+00	1.98E+00
	Min	**3.83E-09**	3.19E-01	2.85E+00	1.93E+01	6.58E+00	2.53E+00	1.27E+00
	Max	**9.48E-07**	6.17E+00	3.82E+00	2.07E+01	1.04E+01	1.29E+01	2.99E+00
	SD	**2.97E-07**	1.27E+00	2.67E-01	3.00E-01	9.95E-01	2.59E+00	4.03E-01
	Sig		+	+	+	+	+	+
500	Mean	**1.49E-02**	1.57E+01	1.90E+01	2.10E+01	1.99E+01	1.69E+01	1.10E+01
	Min	**6.36E-04**	1.50E+01	1.89E+01	2.04E+01	1.99E+01	1.50E+01	9.82E+00
	Max	**9.06E-02**	1.73E+01	1.91E+01	2.11E+01	2.00E+01	1.89E+01	1.29E+01
	SD	**2.05E-02**	5.60E-01	6.38E-02	1.51E-01	4.57E-02	1.00E+00	7.97E-01
	Sig		+	+	+	+	+	+
700	Mean	**5.16E-02**	1.80E+01	1.98E+01	2.11E+01	2.03E+01	1.72E+01	1.29E+01
	Min	**7.01E-05**	1.71E+01	1.97E+01	2.10E+01	2.02E+01	1.46E+01	1.22E+01
	Max	**3.45E-01**	1.86E+01	1.99E+01	2.11E+01	2.04E+01	1.90E+01	1.39E+01
	SD	**9.84E-02**	4.01E-01	3.65E-02	3.72E-02	4.74E-02	1.32E+00	4.94E-01
	Sig		+	+	+	+	+	+
900	Mean	**5.63E-02**	1.91E+01	2.02E+01	2.11E+01	2.05E+01	1.80E+01	1.42E+01
	Min	**2.58E-03**	1.85E+01	2.02E+01	2.10E+01	2.04E+01	1.62E+01	1.34E+01
	Max	**3.46E-01**	1.97E+01	2.02E+01	2.11E+01	2.05E+01	1.90E+01	1.55E+01
	SD	**8.02E-02**	2.80E-01	1.87E-02	2.92E-02	2.44E-02	6.74E-01	4.91E-01
	Sig		+	+	+	+	+	+
		**Griewank**						
100	Mean	**3.46E-13**	9.99E-01	1.05E+00	1.94E+03	2.71E+00	3.86E+01	1.52E-02
	Min	**0.00E+00**	1.68E-01	7.91E-01	3.05E+02	1.08E+00	1.50E+00	1.97E-04
	Max	**3.23E-12**	9.06E+00	1.35E+00	2.23E+03	1.02E+01	1.87E+02	1.29E-01
	SD	**7.53E-13**	2.14E+00	1.14E-01	4.09E+02	2.57E+00	5.62E+01	2.99E-02
	Sig		+	+	+	+	+	+
500	Mean	**2.99E-04**	1.65E+03	3.56E+03	1.33E+04	5.67E+03	4.17E+03	1.70E+02
	Min	**3.98E-10**	1.16E+03	3.24E+03	1.29E+04	4.86E+03	2.08E+03	1.00E+02
	Max	**2.30E-03**	2.97E+03	3.92E+03	1.38E+04	6.32E+03	5.14E+03	2.39E+02
	SD	**5.69E-04**	3.72E+02	1.79E+02	2.54E+02	3.75E+02	8.10E+02	3.38E+01
	Sig		+	+	+	+	+	+
700	Mean	**1.67E-03**	4.40E+03	7.85E+03	1.91E+04	1.07E+04	7.34E+03	5.07E+02
	Min	**6.24E-07**	3.60E+03	7.17E+03	1.81E+04	9.70E+03	3.78E+03	3.56E+02
	Max	**1.98E-02**	5.32E+03	8.28E+03	1.97E+04	1.17E+04	9.11E+03	6.16E+02
	SD	**4.42E-03**	4.60E+02	3.30E+02	3.41E+02	5.68E+02	1.38E+03	6.93E+01
	Sig		+	+	+	+	+	+
900	Mean	**8.18E-03**	8.32E+03	1.29E+04	2.49E+04	1.61E+04	1.04E+04	9.44E+02
	Min	**4.51E-05**	7.34E+03	1.23E+04	2.42E+04	1.46E+04	7.06E+03	7.38E+02
	Max	**3.84E-02**	1.04E+04	1.33E+04	2.56E+04	1.79E+04	1.28E+04	1.09E+03
	SD	**9.96E-03**	6.45E+02	2.79E+02	3.64E+02	7.20E+02	1.72E+03	9.95E+01
	Sig		+	+	+	+	+	+

**Table 7:** Rankings of the algorithms by the Friedman’s test on the basic benchmark functions.

Algorithm	Ranking			
	100*D*	500*D*	700*D*	900*D*
**R-ABC**	**1.0000**	**1.0000**	**1.0000**	**1.0000**
**APABC**	3.5313	2.7812	2.9437	3.1563
**aABC**	3.7000	4.5563	4.8438	4.9875
**ABCVSS**	7.0000	7.0000	7.0000	7.0000
**ABC**	4.1812	5.4875	5.6437	5.6750
**Starling PSO**	5.6312	4.5312	3.9687	3.6625
**JADE**	2.9563	2.6438	2.6000	2.5188

**Table 8:** Results for basic benchmark functions (*MCN* = 10000).

*D*	Stat	R-ABC	BSF-ABC	Random *R*	R-ABC	BSF-ABC	Random *R*
		**Sphere**			**Sum Squares**		
100	Mean	**0.00E+00**	**0.00E+00**	1.99E-141	**0.00E+00**	2.00E-307	1.43E-124
	Min	**0.00E+00**	**0.00E+00**	**0.00E+00**	**0.00E+00**	**0.00E+00**	2.30E-306
	Max	**0.00E+00**	**0.00E+00**	3.98E-140	**0.00E+00**	4.00E-306	2.86E-123
	SD	**0.00E+00**	**0.00E+00**	8.90E-141	**0.00E+00**	**0.00E+00**	6.39E-124
	Sig		**=**	+		**=**	+
500	Mean	**8.44E-194**	2.32E-142	1.17E-47	**1.41E-189**	6.36E-141	1.29E-42
	Min	**1.62E-206**	1.73E-185	1.89E-138	**7.50E-211**	5.46E-183	2.25E-139
	Max	**1.16E-192**	4.64E-141	1.46E-46	**1.54E-188**	1.20E-139	2.58E-41
	SD	**0.00E+00**	1.04E-141	3.72E-47	**0.00E+00**	2.68E-140	5.76E-42
	Sig		+	+		+	+
700	Mean	**2.52E-169**	2.07E-115	4.47E-41	**1.77E-164**	1.53E-123	7.27E-38
	Min	**1.52E-183**	1.53E-165	2.86E-121	**2.88E-181**	3.76E-151	5.63E-103
	Max	**4.96E-168**	2.58E-114	8.90E-40	**2.76E-163**	3.07E-122	1.45E-36
	SD	**0.00E+00**	6.59E-115	1.99E-40	**0.00E+00**	6.86E-123	3.24E-37
	Sig		+	+		+	+
900	Mean	**1.49E-153**	7.55E-111	1.22E-32	**1.70E-153**	1.72E-106	1.46E-23
	Min	**2.08E-169**	2.31E-133	1.73E-90	**1.72E-167**	1.26E-137	4.06E-96
	Max	**2.98E-152**	1.50E-109	2.45E-31	**1.84E-152**	3.44E-105	2.92E-22
	SD	**6.67E-153**	3.35E-110	5.47E-32	**4.78E-153**	7.69E-106	6.52E-23
	Sig		+	+		+	+
		**Dixon-Price**			**Rosenbrock**		
100	Mean	1.18E-01	6.00E-01	**2.44E-02**	1.04E-05	3.05E-03	**1.02E-08**
	Min	1.05E-03	2.56E-03	**1.13E-04**	5.24E-18	**0.00E+00**	1.25E-28
	Max	**2.01E-01**	6.67E-01	2.15E-01	2.08E-04	6.10E-02	**1.33E-07**
	SD	6.50E-02	2.04E-01	**5.46E-02**	4.64E-05	1.36E-02	**3.29E-08**
	Sig		+	**-**		+	-
500	Mean	**2.35E-01**	6.67E-01	6.63E-01	**9.61E-01**	1.06E+00	4.81E+00
	Min	**1.68E-01**	6.67E-01	2.54E-01	5.64E-04	**0.00E+00**	1.36E-04
	Max	**2.52E-01**	6.69E-01	1.01E+00	8.65E+00	**7.39E+00**	3.21E+01
	SD	2.81E-02	**4.44E-04**	3.16E-01	**2.00E+00**	2.39E+00	8.24E+00
	Sig		**+**	+		+	+
700	Mean	**2.45E-01**	6.67E-01	8.62E-01	**2.54E-01**	1.35E+00	4.96E+00
	Min	**1.79E-01**	6.67E-01	2.59E-01	1.75E-04	**2.80E-27**	7.81E-04
	Max	**2.62E-01**	6.67E-01	1.67E+00	**1.96E+00**	8.77E+00	2.07E+01
	SD	2.15E-02	**2.08E-05**	3.82E-01	**4.57E-01**	2.64E+00	5.63E+00
	Sig		**+**	+		=	+
900	Mean	**2.48E-01**	7.31E-01	1.07E+00	**6.12E-01**	2.55E+00	1.03E+01
	Min	**1.90E-01**	6.67E-01	2.56E-01	1.06E-05	**9.82E-26**	1.73E-02
	Max	**2.59E-01**	1.00E+00	2.79E+00	**4.12E+00**	2.99E+01	4.16E+01
	SD	**1.38E-02**	1.24E-01	6.32E-01	**1.04E+00**	6.60E+00	1.42E+01
	Sig		+	+		=	+
* *		**Rastrigin**			**Schwefel**		
100	Mean	**0.00E+00**	**0.00E+00**	**0.00E+00**	**1.27E-03**	**1.27E-03**	**1.27E-03**
	Min	**0.00E+00**	**0.00E+00**	**0.00E+00**	**1.27E-03**	**1.27E-03**	**1.27E-03**
	Max	**0.00E+00**	**0.00E+00**	**0.00E+00**	**1.27E-03**	**1.27E-03**	**1.27E-03**
	SD	**0.00E+00**	**0.00E+00**	**0.00E+00**	**0.00E+00**	**0.00E+00**	**0.00E+00**
	Sig		**=**	**=**		**=**	=
500	Mean	**0.00E+00**	**0.00E+00**	1.31E-05	**6.36E-03**	**6.36E-03**	6.40E-03
	Min	**0.00E+00**	**0.00E+00**	**0.00E+00**	**6.36E-03**	**6.36E-03**	**6.36E-03**
	Max	**0.00E+00**	**0.00E+00**	7.98E-05	**6.36E-03**	**6.36E-03**	6.81E-03
	SD	**0.00E+00**	**0.00E+00**	2.71E-05	**0.00E+00**	**0.00E+00**	1.08E-04
	Sig		**=**	+		=	+
700	Mean	**0.00E+00**	**0.00E+00**	5.90E-04	**8.91E-03**	**8.91E-03**	9.63E-03
	Min	**0.00E+00**	**0.00E+00**	6.69E-08	**8.91E-03**	**8.91E-03**	**8.91E-03**
	Max	**0.00E+00**	**0.00E+00**	4.63E-03	**8.91E-03**	**8.91E-03**	1.24E-02
	SD	**0.00E+00**	**0.00E+00**	1.15E-03	**0.00E+00**	**0.00E+00**	8.52E-04
	Sig		**=**	+		=	+
900	Mean	**0.00E+00**	**0.00E+00**	7.51E-03	**1.15E-02**	**1.15E-02**	2.55E-02
	Min	**0.00E+00**	**0.00E+00**	7.94E-07	**1.15E-02**	**1.15E-02**	**1.15E-02**
	Max	**0.00E+00**	**0.00E+00**	5.58E-02	**1.15E-02**	**1.15E-02**	8.42E-02
	SD	**0.00E+00**	**0.00E+00**	1.42E-02	1.30E-11	**0.00E+00**	2.08E-02
	Sig		**=**	+		**=**	+
* *		**Ackley**			**Griewank**		
100	Mean	**8.88E-16**	**8.88E-16**	2.66E-15	**0.00E+00**	**0.00E+00**	**0.00E+00**
	Min	**8.88E-16**	**8.88E-16**	**8.88E-16**	**0.00E+00**	**0.00E+00**	**0.00E+00**
	Max	**8.88E-16**	**8.88E-16**	7.99E-15	**0.00E+00**	**0.00E+00**	**0.00E+00**
	SD	**0.00E+00**	**0.00E+00**	2.70E-15	**0.00E+00**	**0.00E+00**	**0.00E+00**
	Sig		**=**	+		**=**	=
500	Mean	**8.88E-16**	**8.88E-16**	1.34E-13	**0.00E+00**	**0.00E+00**	**0.00E+00**
	Min	**8.88E-16**	**8.88E-16**	7.99E-15	**0.00E+00**	**0.00E+00**	**0.00E+00**
	Max	**8.88E-16**	**8.88E-16**	5.23E-13	**0.00E+00**	**0.00E+00**	**0.00E+00**
	SD	**0.00E+00**	**0.00E+00**	1.61E-13	**0.00E+00**	**0.00E+00**	**0.00E+00**
	Sig		**=**	+		**=**	=
700	Mean	**8.88E-16**	**8.88E-16**	2.61E-13	**0.00E+00**	**0.00E+00**	**0.00E+00**
	Min	**8.88E-16**	**8.88E-16**	2.22E-14	**0.00E+00**	**0.00E+00**	**0.00E+00**
	Max	**8.88E-16**	**8.88E-16**	1.06E-12	**0.00E+00**	**0.00E+00**	**0.00E+00**
	SD	**0.00E+00**	**0.00E+00**	2.76E-13	**0.00E+00**	**0.00E+00**	**0.00E+00**
	Sig		**=**	+		**=**	=
900	Mean	**8.88E-16**	**8.88E-16**	3.15E-12	**0.00E+00**	**0.00E+00**	8.33E-17
	Min	**8.88E-16**	**8.88E-16**	1.04E-13	**0.00E+00**	**0.00E+00**	**0.00E+00**
	Max	**8.88E-16**	**8.88E-16**	4.55E-11	**0.00E+00**	**0.00E+00**	1.11E-15
	SD	**0.00E+00**	**0.00E+00**	1.00E-11	**0.00E+00**	**0.00E+00**	2.72E-16
	Sig		**=**	+		**=**	=

Table 6 shows that, with 30000 fitness evaluations, the R-ABC algorithm gives final solutions significantly better than the APABC, aABC, ABC, Starling PSO, and JADE algorithms for the unimodal separable, unimodal non-separable, multi-modal separable, and multi-modal non-separable benchmark functions for all tested dimensions. The rankings of all compared algorithms for the basic benchmark functions are shown in Table 7. The R-ABC algorithm is the first ranked among all competitors for all tested dimensions. The JADE algorithm is the second ranked for the majority of cases.

Table 8 shows that, with *MCN* = 10000, the R-ABC algorithm gives solutions significantly better than the BSF-ABC algorithm in the majority of cases of the unimodal separable and unimodal non-separable functions. Both the R-ABC and BSF-ABC algorithms can reach the global optimum solutions for all runs of the 100*D* Sphere function, but only the R-ABC algorithm can reach the global optimum solutions for all runs of the 100*D* Sum Squares function. According to the Wilcoxon’s rank sum test, the solutions of the R-ABC algorithm are similar to those of the BSF-ABC algorithm for the 100*D* Sphere function, the 100*D* Sum Squares function, and the 700*D* and 900*D* Rosenbrock functions. For the multi-modal separable and multi-modal non-separable functions, the solutions of the R-ABC algorithm are similar to those of the BSF-ABC algorithm.

The result also shows the different sensitivity to the number of dimensions. The growing number of dimensions affects the solution quality of the R-ABC algorithm less than those of some algorithms. This is obvious in the cases of the Sphere and Sum Squares functions. Table 8 shows that both the R-ABC and BSF-ABC algorithms can reach the global optimum solutions for all runs of the 100*D* Sphere function with *MCN* = 10000, but the BSF-ABC algorithm was beaten by the R-ABC algorithm for 500*D*, 700*D*, and 900*D*. In Table 6, for the Sphere and Sum Squares functions, with *MFE* = 30000, the aABC algorithm provides the third-best mean solutions for 100*D*, but it provides the fifth-best mean solutions for 900*D*, while the R-ABC algorithm provides the best mean solutions for both 100*D* and 900*D*. This shows that the mechanism of the R-ABC algorithm is not sensitive to the number of dimensions, so it also works well with the high-dimensional basic benchmark functions.

The experiments were designed to evaluate the R-ABC algorithm on four categories of benchmark functions. We found that the different categories of benchmark functions did not affect the R-ABC’s quality in any consistent way. Table 8 shows that the R-ABC algorithm can reach the global optimum solutions for all tested dimensions of the Rastrigin, Ackley, and Griewank functions and for the 100*D* Sphere and Sum Squares function within *MCN* = 10000. Reaching the global optimum solutions for the Ackley and Griewank functions, which are multimodal and non-separable, shows that the difficulty of solving multimodal functions and that of solving separable functions do not affect the performance of the R-ABC algorithm in this case. However, Table 6 shows that the JADE and APABC algorithms performed well in some categories of benchmark functions. For all tested dimensions of the unimodal separable and unimodal non-separable functions, the JADE algorithm gives the second-best solutions. For the multi-modal separable functions, the APABC algorithm gives the second-best mean solutions in all cases, except for the 900*D* Rastrigin function. For the multi-modal non-separable functions, the JADE algorithm gives the second-best mean solutions in all cases, except for the 100*D* Ackley function.

The solution quality of the R-ABC algorithm is rather affected by the surface of the benchmark functions. The surfaces of the Rastrigin, Ackley, and Griewank functions are like big mountains. The highest point is at the center and surrounded by smaller peaks roughly in descending order. The best food source found so far in Eq (11) guides onlooker bees to a higher step of the mountain.

However, the R-ABC is not totally superior to the BSF-ABC algorithm for the Rosenbrock function which is unimodal. For all tested dimensions of the Rosenbrock function, the R-ABC algorithm provides mean solutions better than the BSF-ABC algorithm, while the BSF-ABC algorithm provides minimum solutions better than the R-ABC algorithm. There is a flat valley around the optimum point on the surface of the Rosenbrock function. When the R-ABC almost converges towards the optimum solution, food sources are likely located in the flat valley including the best food source found. The short distance between the best food source found and each food source makes it difficult for the R-ABC algorithm to reach the optimum solution.

In addition, to validate the effectiveness of the reinforcement vectors on each benchmark function, we replaced the reinforcement vectors with random vectors. Table 8 also shows the results of the reinforcement vectors compared to those of random vectors. The R-ABC algorithm gives solutions significantly better than the algorithm with random vectors in the majority of cases. The R-ABC algorithm is beaten by the algorithm with random vectors for the 100*D* Dixon-Price function and the 100*D* Rosenbrock function. In the case of the Rosenbrock function, the R-ABC algorithm gets into difficulties when it adapts the values of the reinforcement vectors in a flat valley surface where the fitness values of the food sources are similar. In the case of the Dixon-Price function, the optimum values of some dimensions are far from those of others, and those values may be out of the range of the reinforcement vectors, which were designed to find a better solution near the current solution.

**Fig 5:**
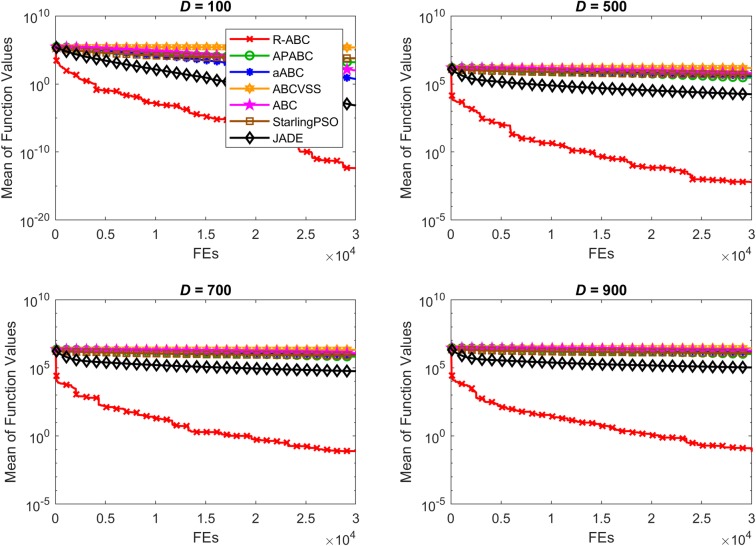
Convergence performance on the Sphere function with different dimensions.

**Fig 6:**
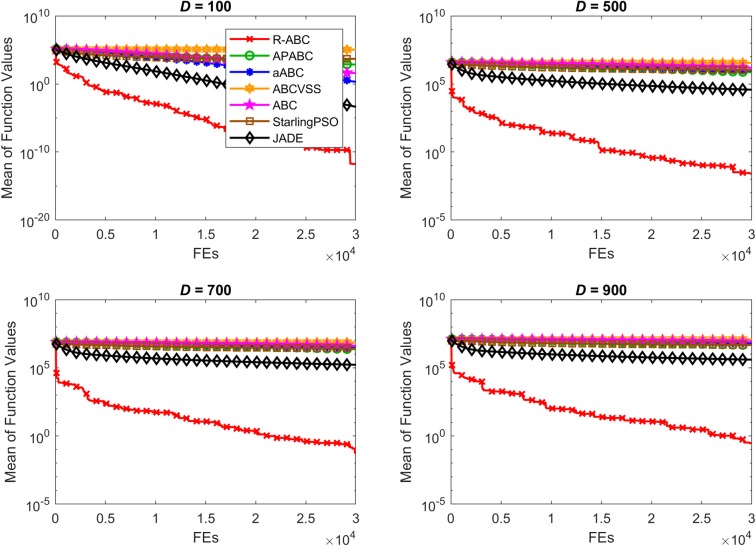
Convergence performance on the Sum Squares function with different dimensions.

**Fig 7:**
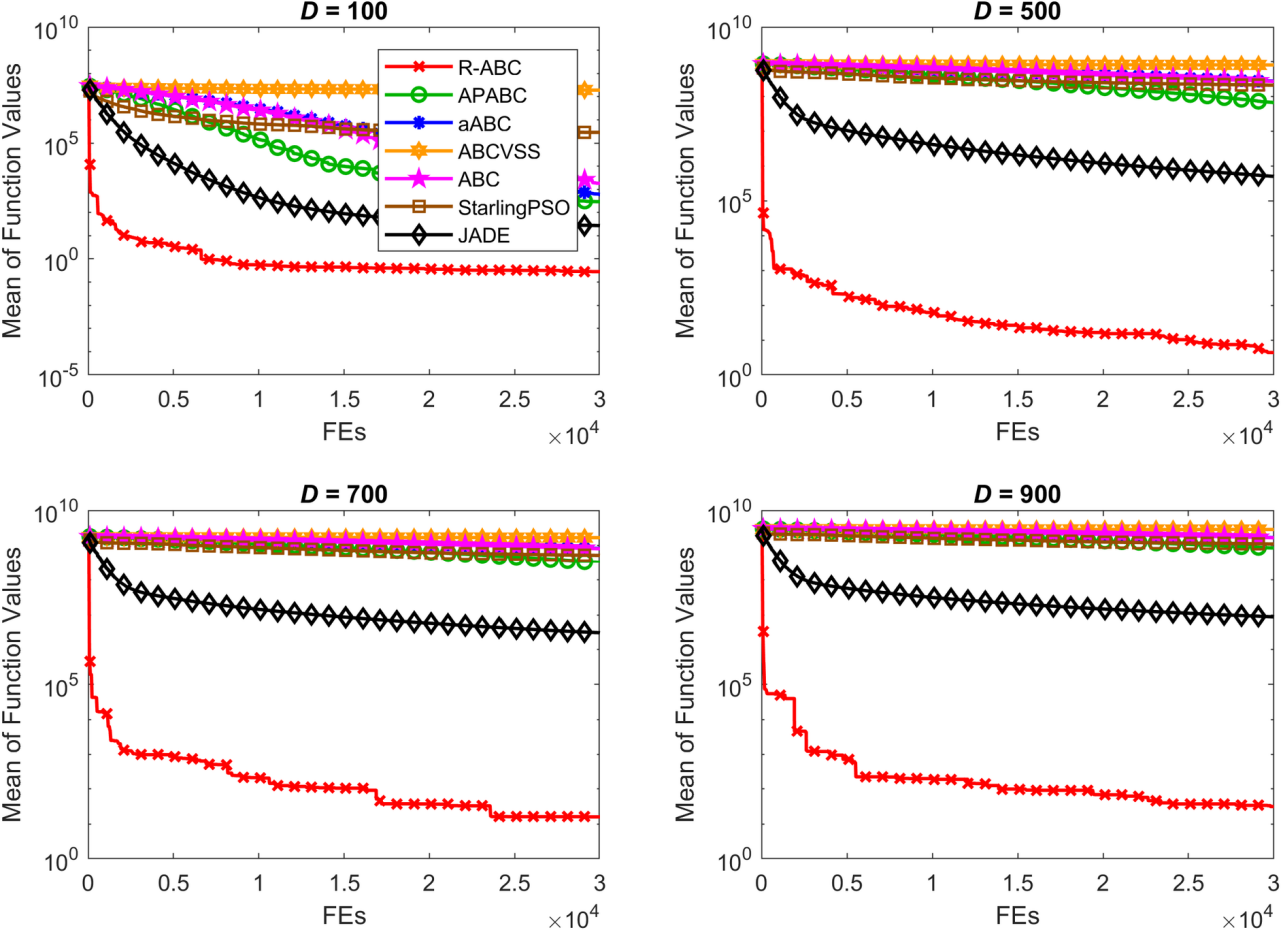
Convergence performance on the Dixon-Price function with different dimensions.

**Fig 8:**
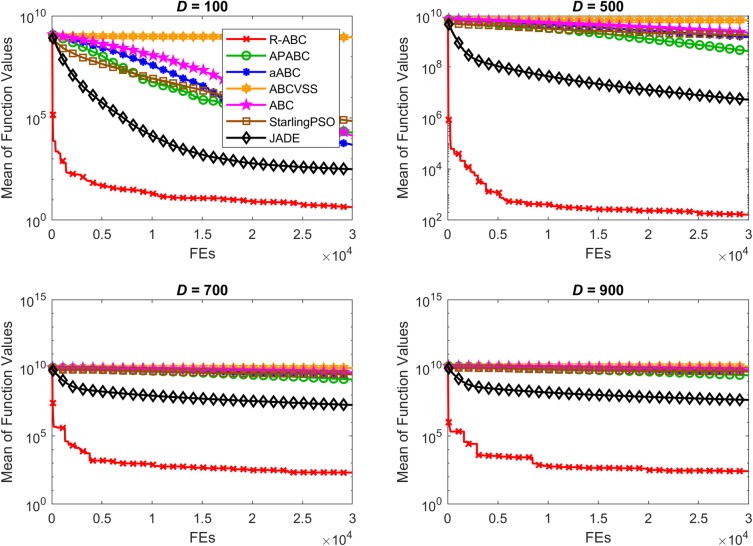
Convergence performance on the Rosenbrock function with different dimensions.

**Fig 9:**
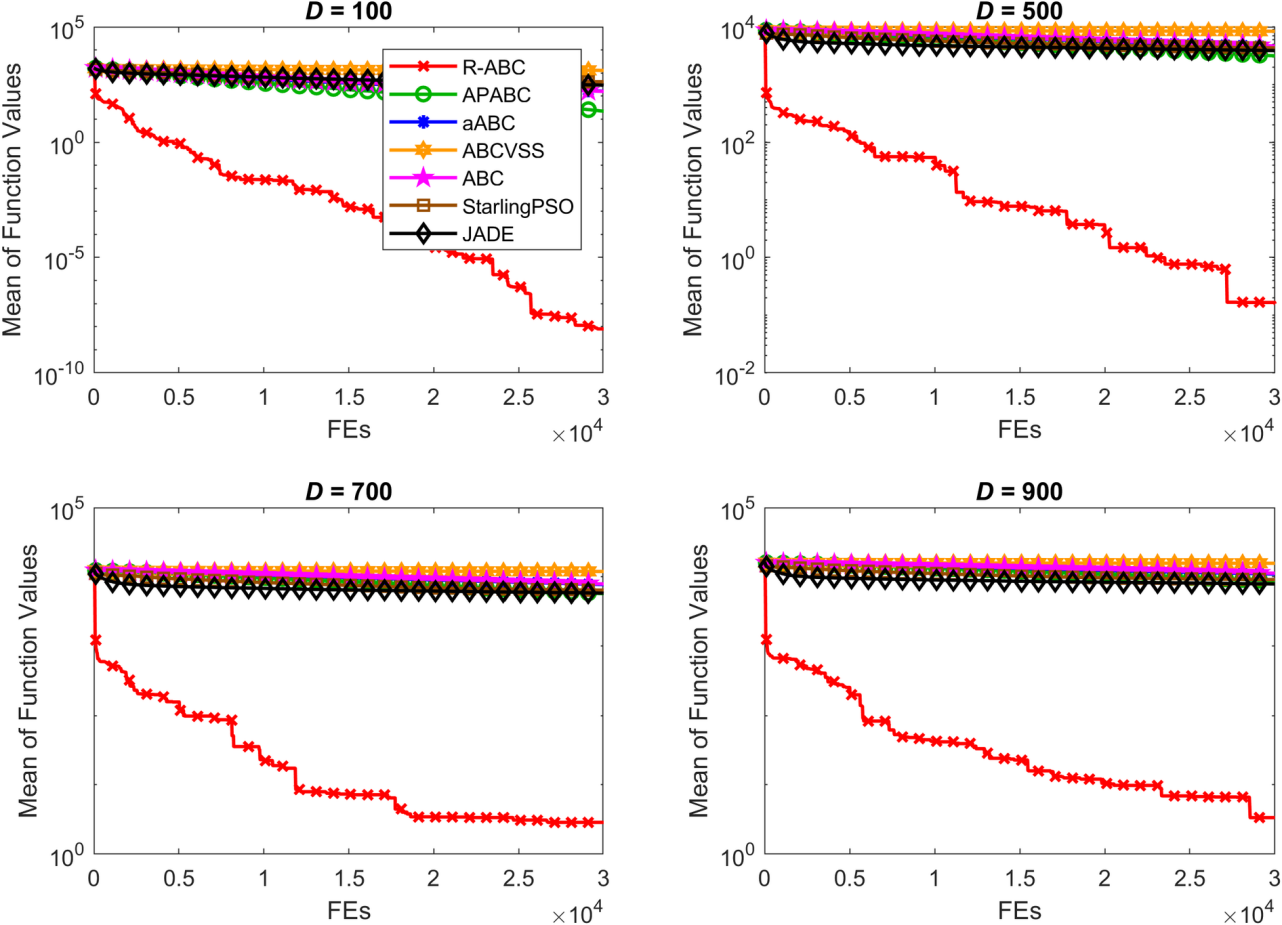
Convergence performance on the Rastrigin function with different dimensions.

**Fig 10:**
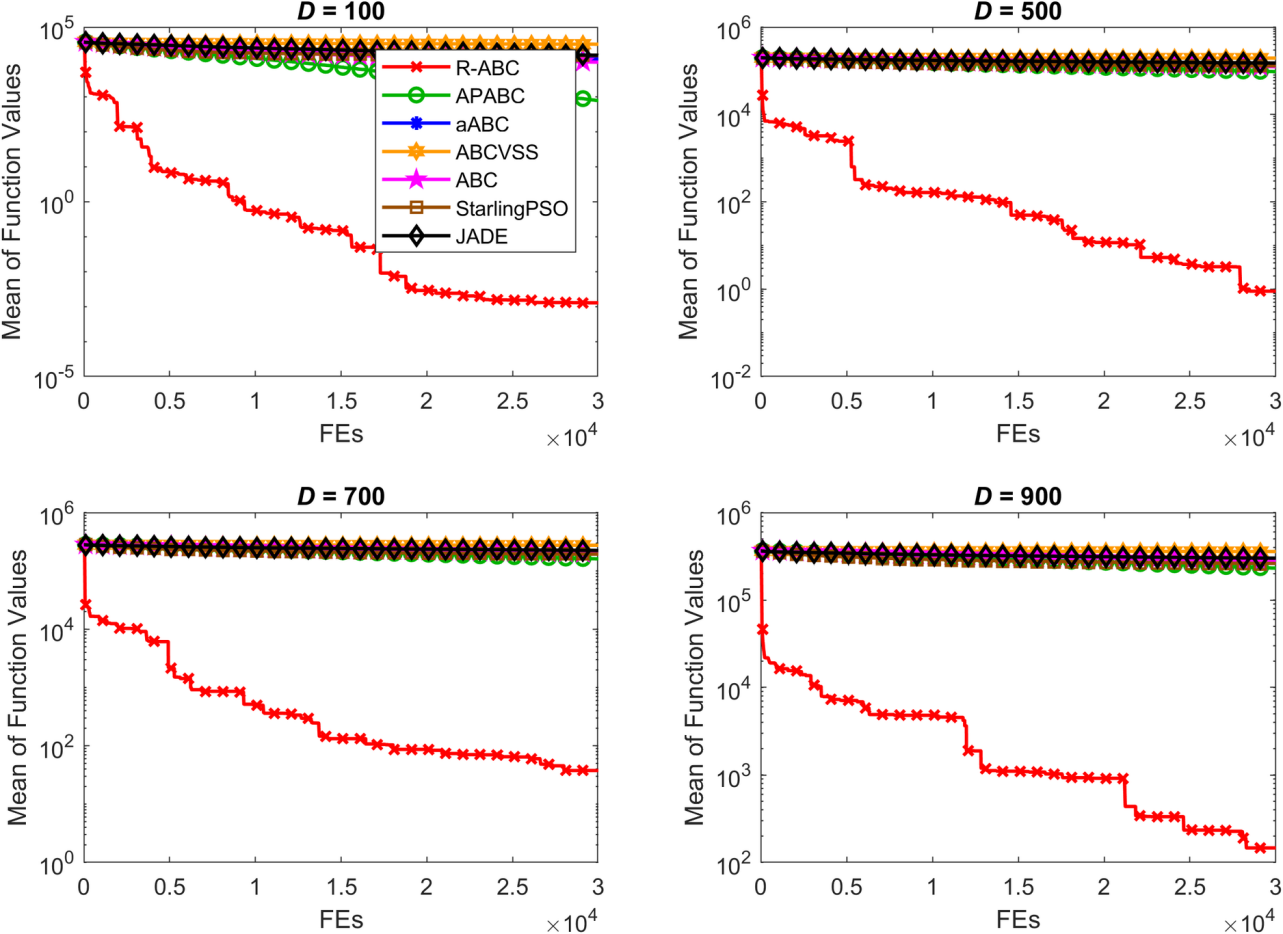
Convergence performance on the Schwefel function with different dimensions.

**Fig 11:**
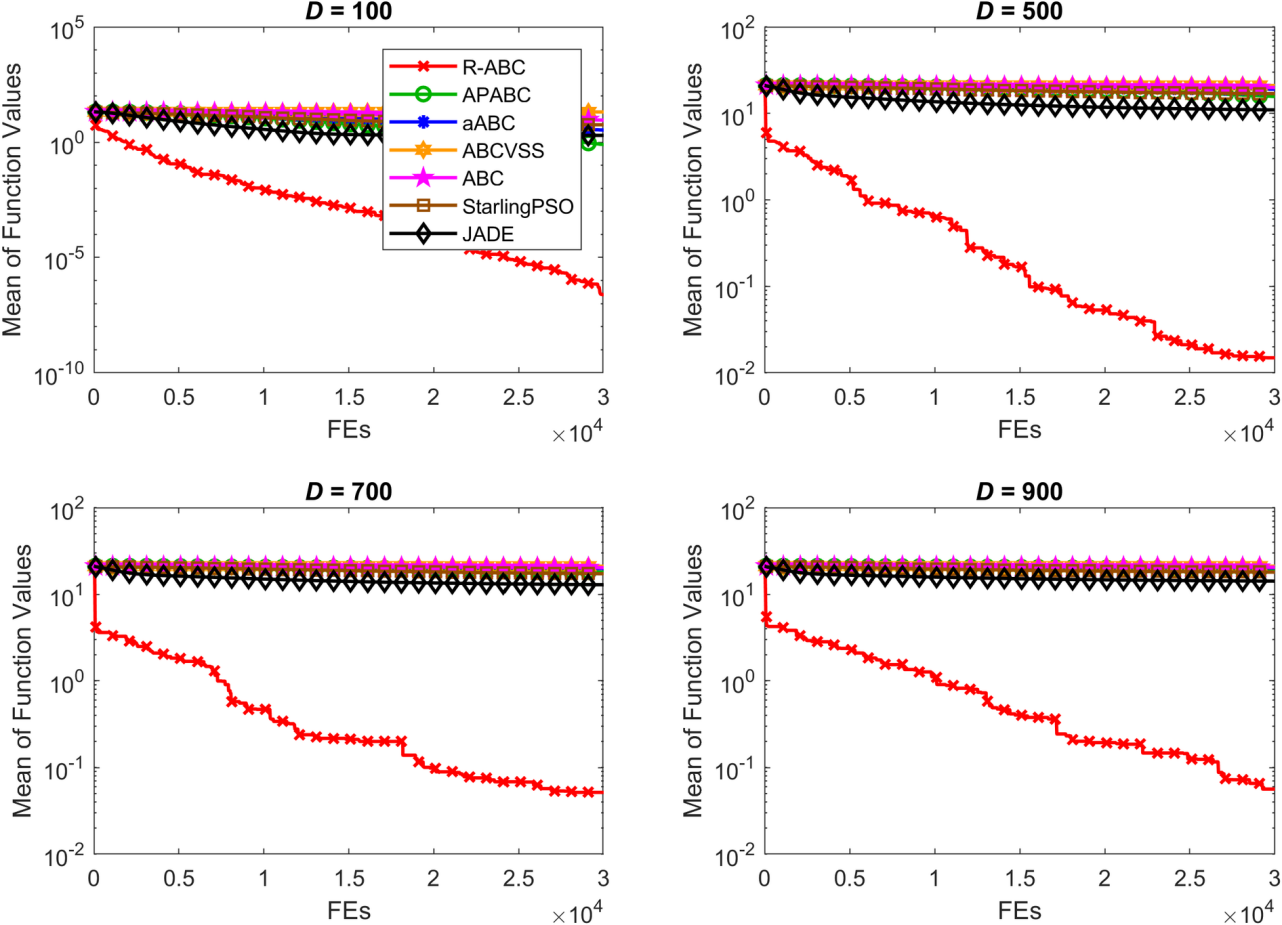
Convergence performance on the Ackley function with different dimensions.

**Fig 12:**
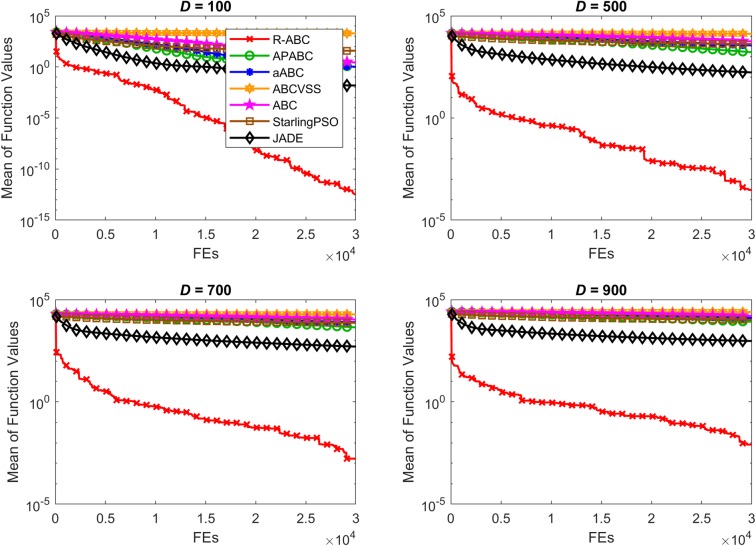
Convergence performance on the Griewank function with different dimensions.

To compare the convergence speed of the algorithms, the results of all benchmark functions are displayed in Figs 5–12. Each contour shows the average of the best solution found. All Y-axes are displayed in log scale. Figs 5 and 6 show that, for the unimodal separable benchmark functions, the Sphere, and Sum Squares functions, the convergence speed of the R-ABC algorithm is faster than the JADE algorithm and far faster than all other algorithms. Figs 7 and 8 show that, for the unimodal non-separable benchmark functions, the Dixon-Price and Rosenbrock functions, the R-ABC initially converge more quickly than others. For the 100*D* unimodal non-separable functions, the Starling PSO algorithm initially converges quickly than the APABC, ABC, and aABC algorithms, but it stagnates too quickly. Figs 9–12 show that, for the multi-modal functions, the contour of the R-ABC algorithm suddenly drops several times, but it can escape from local optimum solutions.

### CEC2005’s shifted functions

**Fig 13:**
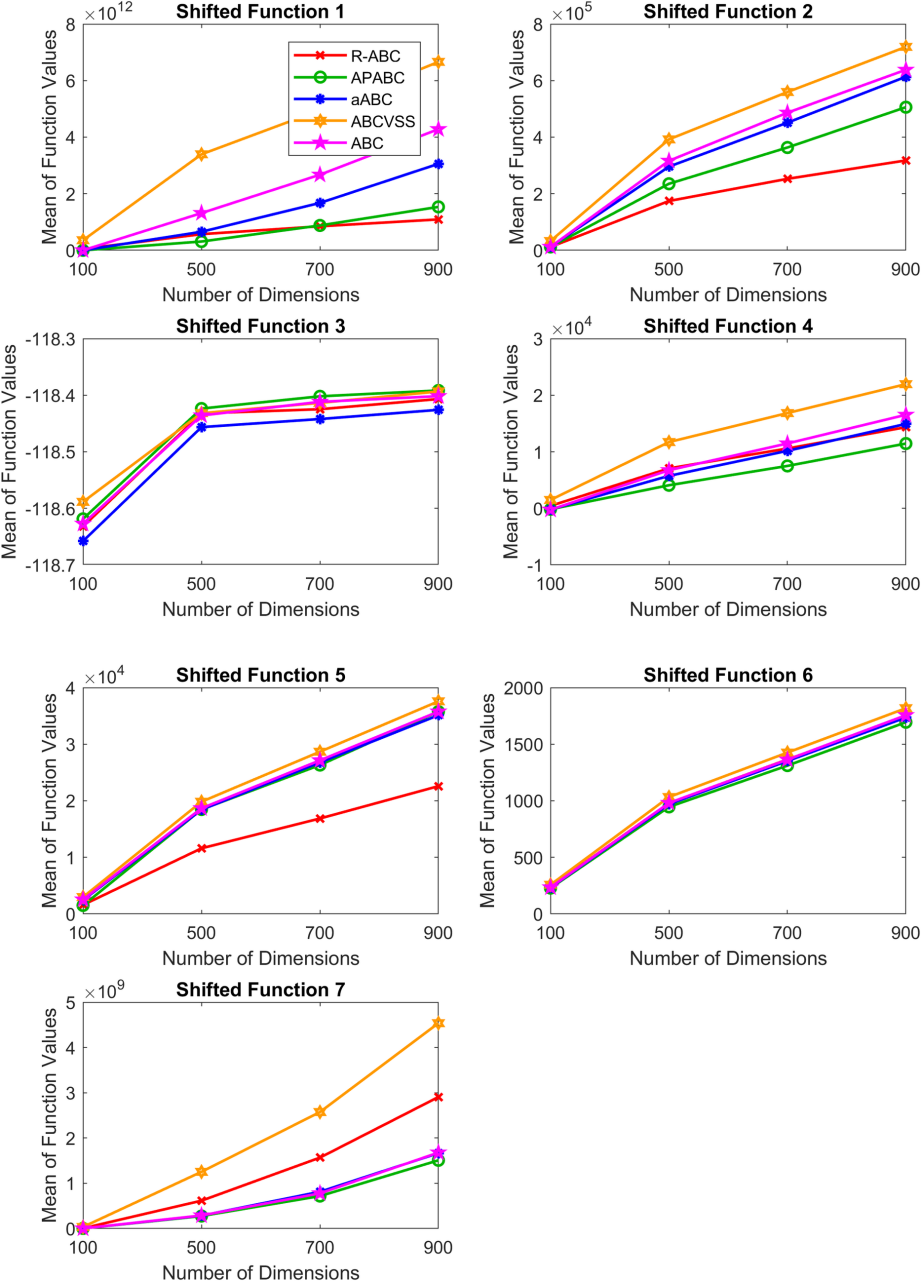
The final results of the CEC2005’s shifted functions for all tested dimensions.

**Fig 14:**
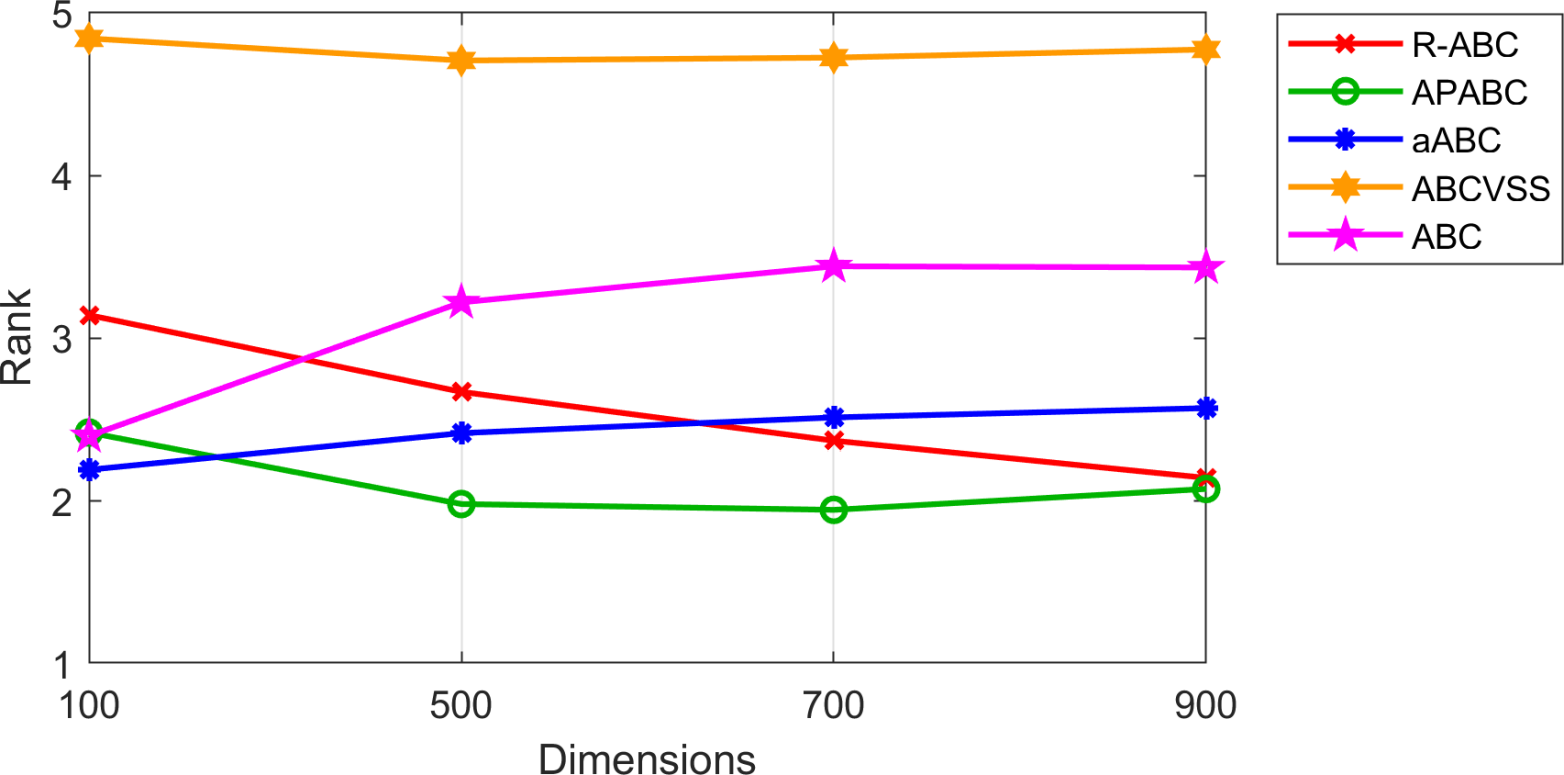
Rankings of the algorithms by the Friedman’s test on the CEC2005’s shifted functions.

To evaluate the performance of the R-ABC algorithm on benchmark functions with more difficulty, we compared the R-ABC algorithm with the original ABC algorithm and its variants which were the APABC, aABC, and ABCVSS algorithms on seven CEC2005’s shifted functions [21] for 100, 500, 700, and 900 dimensions. Table 9 shows the results on the CEC2005’s shifted functions. Table 10 shows the results of the Wilcoxon’s rank sum test for each dimension. Fig 13 shows the final results for all tested dimensions. Fig 14 shows the results of the algorithm ranking by the Friedman’s test by using KEEL [25–26]. Note that the tested dimensions are 100, 500, 700, and 900, so there are irregular intervals of x-axis between 100 and 500 in Figs 13 and 14.

**Table 9:** Results for CEC2005’s shifted functions (*MFE* = 30000).

*D*	Stat	R-ABC	APABC	aABC	ABCVSS	ABC
		Shifted Function 1				
100	Mean	4.66E+10	4.24E+07	**1.32E+05**	3.56E+11	4.16E+06
	Min	3.64E+10	1.08E+07	1.20E+04	9.67E+10	**1.08E+04**
	Max	5.42E+10	9.40E+07	**1.17E+06**	4.66E+11	8.03E+07
	SD	5.32E+09	2.59E+07	**2.65E+05**	8.12E+10	1.79E+07
	Sig		-	-	+	-
500	Mean	5.71E+11	**3.11E+11**	6.55E+11	3.39E+12	1.32E+12
	Min	5.06E+11	**1.81E+11**	5.66E+11	3.01E+12	1.05E+12
	Max	6.43E+11	**4.93E+11**	8.00E+11	3.62E+12	1.70E+12
	SD	**3.59E+10**	9.08E+10	5.55E+10	1.47E+11	1.56E+11
	Sig		-	+	+	+
700	Mean	**8.50E+11**	8.79E+11	1.68E+12	4.95E+12	2.67E+12
	Min	7.61E+11	5.37E+11	1.49E+12	4.25E+12	1.92E+12
	Max	**9.60E+11**	1.60E+12	1.88E+12	5.35E+12	2.95E+12
	SD	**5.07E+10**	2.90E+11	1.07E+11	2.31E+11	2.29E+11
	Sig		=	+	+	+
900	Mean	**1.09E+12**	1.53E+12	3.06E+12	6.66E+12	4.28E+12
	Min	**1.01E+12**	1.11E+12	2.59E+12	6.17E+12	3.89E+12
	Max	**1.14E+12**	1.96E+12	3.46E+12	7.21E+12	4.79E+12
	SD	**4.25E+10**	2.45E+11	2.39E+11	2.75E+11	2.44E+11
	Sig		+	+	+	+
		**Shifted Function 2**				
100	Mean	**1.19E+04**	1.19E+04	1.27E+04	3.29E+04	1.22E+04
	Min	**8.38E+03**	9.21E+03	1.10E+04	1.29E+04	8.40E+03
	Max	1.58E+04	1.55E+04	1.57E+04	4.25E+04	**1.50E+04**
	SD	1.85E+03	1.55E+03	**1.46E+03**	6.52E+03	1.68E+03
	Sig		=	=	+	=
500	Mean	**1.74E+05**	2.35E+05	2.96E+05	3.92E+05	3.16E+05
	Min	**1.61E+05**	2.14E+05	2.67E+05	3.61E+05	2.79E+05
	Max	**1.88E+05**	2.62E+05	3.35E+05	4.25E+05	3.60E+05
	SD	**7.74E+03**	1.28E+04	1.60E+04	1.74E+04	1.94E+04
	Sig		+	+	+	+
700	Mean	**2.53E+05**	3.63E+05	4.51E+05	5.60E+05	4.87E+05
	Min	**2.24E+05**	3.30E+05	4.12E+05	5.10E+05	4.48E+05
	Max	**2.79E+05**	3.91E+05	4.87E+05	5.92E+05	5.22E+05
	SD	**1.26E+04**	1.78E+04	2.17E+04	2.50E+04	2.10E+04
	Sig		+	+	+	+
900	Mean	**3.18E+05**	5.06E+05	6.14E+05	7.19E+05	6.38E+05
	Min	**2.94E+05**	4.62E+05	5.81E+05	6.49E+05	5.96E+05
	Max	**3.35E+05**	5.87E+05	6.73E+05	7.72E+05	6.84E+05
	SD	**1.17E+04**	3.06E+04	2.71E+04	3.19E+04	2.36E+04
	Sig		+	+	+	+
		**Shifted Function 3**				
100	Mean	-1.19E+02	-1.19E+02	**-1.19E+02**	-1.19E+02	-1.19E+02
	Min	-1.19E+02	-1.19E+02	**-1.19E+02**	-1.19E+02	-1.19E+02
	Max	-1.19E+02	-1.19E+02	**-1.19E+02**	-1.18E+02	-1.19E+02
	SD	3.01E-02	2.58E-02	4.71E-02	4.42E-02	**2.39E-02**
	Sig		=	=	=	=
500	Mean	-1.18E+02	-1.18E+02	**-1.18E+02**	-1.18E+02	-1.18E+02
	Min	-1.18E+02	-1.18E+02	**-1.18E+02**	-1.18E+02	-1.18E+02
	Max	-1.18E+02	-1.18E+02	**-1.18E+02**	-1.18E+02	-1.18E+02
	SD	1.53E-02	**1.10E-02**	1.43E-02	2.13E-02	1.30E-02
	Sig		=	=	=	=
700	Mean	-1.18E+02	-1.18E+02	**-1.18E+02**	-1.18E+02	-1.18E+02
	Min	**-1.19E+02**	-1.18E+02	-1.18E+02	-1.18E+02	-1.18E+02
	Max	-1.18E+02	-1.18E+02	**-1.18E+02**	-1.18E+02	-1.18E+02
	SD	3.42E-02	7.56E-03	1.55E-02	1.84E-02	**7.05E-03**
	Sig		=	=	=	=
900	Mean	-1.18E+02	-1.18E+02	**-1.18E+02**	-1.18E+02	-1.18E+02
	Min	**-1.18E+02**	-1.18E+02	-1.18E+02	-1.18E+02	-1.18E+02
	Max	-1.18E+02	-1.18E+02	**-1.18E+02**	-1.18E+02	-1.18E+02
	SD	2.54E-02	**9.06E-03**	1.79E-02	1.45E-02	1.09E-02
	Sig		=	=	=	=
		**Shifted Function 4**				
100	Mean	4.41E+02	-1.62E+02	-1.95E+02	1.48E+03	**-2.32E+02**
	Min	3.85E+02	-2.10E+02	-2.18E+02	7.85E+02	**-2.73E+02**
	Max	4.72E+02	-1.28E+02	-1.62E+02	1.83E+03	**-1.82E+02**
	SD	2.35E+01	1.95E+01	**1.47E+01**	2.67E+02	2.35E+01
	Sig		-	-	+	-
500	Mean	7.06E+03	**4.08E+03**	5.74E+03	1.17E+04	6.74E+03
	Min	6.55E+03	**3.50E+03**	5.44E+03	1.11E+04	6.11E+03
	Max	7.43E+03	**4.66E+03**	6.06E+03	1.20E+04	7.69E+03
	SD	2.27E+02	3.31E+02	**1.52E+02**	2.21E+02	3.34E+02
	Sig		-	-	+	-
700	Mean	1.06E+04	**7.50E+03**	1.02E+04	1.68E+04	1.15E+04
	Min	1.01E+04	**6.74E+03**	9.55E+03	1.63E+04	1.03E+04
	Max	1.09E+04	**8.24E+03**	1.06E+04	1.73E+04	1.23E+04
	SD	**1.95E+02**	4.41E+02	2.56E+02	3.06E+02	5.13E+02
	Sig		-	-	+	+
900	Mean	1.43E+04	**1.15E+04**	1.49E+04	2.20E+04	1.65E+04
	Min	1.39E+04	**1.03E+04**	1.41E+04	2.12E+04	1.56E+04
	Max	1.49E+04	**1.32E+04**	1.55E+04	2.25E+04	1.71E+04
	SD	**2.77E+02**	6.53E+02	3.23E+02	3.24E+02	4.41E+02
	Sig		-	+	+	+
		**Shifted Function 5**				
100	Mean	1.59E+03	1.50E+03	**2.44E+03**	2.98E+03	2.54E+03
	Min	1.31E+03	1.31E+03	**2.20E+03**	2.64E+03	2.16E+03
	Max	1.78E+03	1.67E+03	**2.74E+03**	3.47E+03	2.74E+03
	SD	1.22E+02	1.03E+02	**1.53E+02**	2.16E+02	1.74E+02
	Sig		-	+	+	+
500	Mean	**1.16E+04**	1.85E+04	1.84E+04	1.98E+04	1.87E+04
	Min	**1.07E+04**	1.65E+04	1.72E+04	1.86E+04	1.77E+04
	Max	**1.25E+04**	2.00E+04	1.94E+04	2.10E+04	1.98E+04
	SD	**4.88E+02**	8.17E+02	6.87E+02	6.52E+02	5.99E+02
	Sig		+	+	+	+
700	Mean	**1.69E+04**	2.63E+04	2.68E+04	2.87E+04	2.71E+04
	Min	**1.64E+04**	2.49E+04	2.48E+04	2.67E+04	2.58E+04
	Max	**1.76E+04**	2.85E+04	2.82E+04	3.02E+04	2.86E+04
	SD	**3.09E+02**	8.69E+02	8.05E+02	8.61E+02	7.03E+02
	Sig		+	+	+	+
900	Mean	**2.26E+04**	3.58E+04	3.51E+04	3.76E+04	3.58E+04
	Min	**2.12E+04**	3.36E+04	3.38E+04	3.54E+04	3.36E+04
	Max	**2.37E+04**	3.76E+04	3.68E+04	3.95E+04	3.72E+04
	SD	**7.69E+02**	1.07E+03	8.83E+02	1.03E+03	9.96E+02
	Sig		+	+	+	+
		**Shifted Function 6**				
100	Mean	2.38E+02	**2.28E+02**	2.32E+02	2.58E+02	2.35E+02
	Min	2.27E+02	**2.22E+02**	2.24E+02	2.49E+02	2.23E+02
	Max	2.47E+02	**2.37E+02**	2.39E+02	2.65E+02	2.40E+02
	SD	4.79E+00	3.76E+00	**3.44E+00**	3.79E+00	3.65E+00
	Sig		-	-	+	-
500	Mean	9.81E+02	**9.46E+02**	9.70E+02	1.03E+03	9.81E+02
	Min	9.60E+02	**9.17E+02**	9.43E+02	1.01E+03	9.64E+02
	Max	9.97E+02	**9.72E+02**	9.83E+02	1.05E+03	9.92E+02
	SD	1.01E+01	1.47E+01	9.72E+00	1.16E+01	**9.29E+00**
	Sig		-	-	+	=
700	Mean	1.37E+03	**1.31E+03**	1.35E+03	1.43E+03	1.36E+03
	Min	1.34E+03	**1.28E+03**	1.33E+03	1.41E+03	1.34E+03
	Max	1.39E+03	**1.33E+03**	1.37E+03	1.45E+03	1.38E+03
	SD	1.26E+01	1.62E+01	**9.75E+00**	1.01E+01	1.00E+01
	Sig		-	-	+	=
900	Mean	1.74E+03	**1.69E+03**	1.74E+03	1.82E+03	1.76E+03
	Min	1.71E+03	**1.66E+03**	1.71E+03	1.79E+03	1.73E+03
	Max	1.77E+03	**1.73E+03**	1.75E+03	1.83E+03	1.78E+03
	SD	1.42E+01	1.97E+01	**1.08E+01**	1.60E+01	1.21E+01
	Sig		-	=	+	+
		**Shifted Function 7**				
100	Mean	5.41E+06	2.70E+06	**2.17E+06**	3.53E+07	2.33E+06
	Min	5.00E+06	2.41E+06	1.69E+06	1.33E+07	**1.67E+06**
	Max	6.24E+06	3.22E+06	**2.47E+06**	4.93E+07	2.69E+06
	SD	3.08E+05	**2.38E+05**	2.69E+05	1.09E+07	3.09E+05
	Sig		-	-	+	-
500	Mean	6.16E+08	**2.76E+08**	2.85E+08	1.25E+09	2.88E+08
	Min	5.63E+08	**2.34E+08**	2.50E+08	8.43E+08	2.50E+08
	Max	6.64E+08	3.31E+08	**3.06E+08**	1.49E+09	3.32E+08
	SD	2.67E+07	2.43E+07	**1.51E+07**	1.48E+08	2.03E+07
	Sig		-	-	+	-
700	Mean	1.57E+09	**7.18E+08**	8.13E+08	2.57E+09	7.78E+08
	Min	1.42E+09	**6.03E+08**	7.15E+08	1.92E+09	6.55E+08
	Max	1.73E+09	9.76E+08	**8.69E+08**	2.88E+09	8.75E+08
	SD	7.23E+07	8.09E+07	**4.34E+07**	2.72E+08	5.39E+07
	Sig		-	-	+	-
900	Mean	2.91E+09	**1.51E+09**	1.67E+09	4.54E+09	1.68E+09
	Min	2.70E+09	**1.28E+09**	1.44E+09	4.15E+09	1.39E+09
	Max	3.10E+09	1.83E+09	**1.80E+09**	4.82E+09	1.84E+09
	SD	1.01E+08	1.27E+08	**8.52E+07**	1.86E+08	1.11E+08
	Sig		-	-	+	-

**Table 10:** The result of Wilcoxon’s test of the algorithm on the CEC2005’s shifted functions.

R-ABC vs	100*D*			500*D*			700*D*			900*D*		
	-	=	+	-	=	+	-	=	+	-	=	+
APABC	5	2	0	4	1	2	3	2	2	3	1	3
aABC	4	2	1	3	1	3	3	1	3	1	2	4
ABCVSS	0	1	6	0	1	6	0	1	6	0	1	6
ABC	4	2	1	2	2	3	1	2	4	1	1	5

Table 9 shows that, with 30000 fitness evaluations, the R-ABC algorithm gives the best solutions on one function for 100 dimensions, two functions for 500 dimensions, three functions for 700 dimensions, and three functions for 900 dimensions. For the Shifted Function 7, the R-ABC algorithm can give the best solutions for all tested dimensions.

The results of the Wilcoxon’s rank sum test in Table 10 show that the number of solutions provided by the R-ABC algorithm which are significantly better than those of the compared algorithms increases when the number of dimensions increases. For 100 dimensions, the numbers of the shifted functions on which the R-ABC algorithm gives solutions significantly better than the APABC, aABC, ABCVSS, and ABC algorithms are 0, 1, 6, and 1, respectively. However, for 900 dimensions, the numbers of the shifted functions on which the R-ABC algorithm gives solutions significantly better than the APABC, aABC, ABCVSS, and ABC algorithms increase to 3, 4, 6, and 5, respectively.

Fig 13 shows the final results of each algorithm for all tested dimensions to compare the trend of the solution quality. The R-ABC provides flatter upward tilts to the lines in the cases of the Shifted Function 1, 2, 3, 4, and 5 compared with other algorithms. This is evidence that the R-ABC algorithm is less sensitive to the increasing number of dimensions than other algorithms for the majority of cases.

Fig 14 shows the rankings of all compared algorithms for the CEC2005’s shifted functions. The aABC algorithm is the first ranked for 100 dimensions, and the APABC algorithm is the fisrt ranked for 500, 700, and 900 dimensions. Although the R-ABC algorithm is not the first ranked for any tested dimension, it is obvious that, when the number of dimensions increases, the R-ABC algorithm gets a better rank while the aABC and ABC algorithms get worse ranks.

### CEC2014’s hybrid functions

To evaluate the performance of the R-ABC algorithm on more complicated functions, we also compared the proposed algorithm with the APABC, aABC, ABCVSS, ABC, Starling PSO, and JADE algorithms on CEC2014’s hybrid functions. Each hybrid function is made up of different basic functions resulting in different subcomponents.

**Fig 15:**
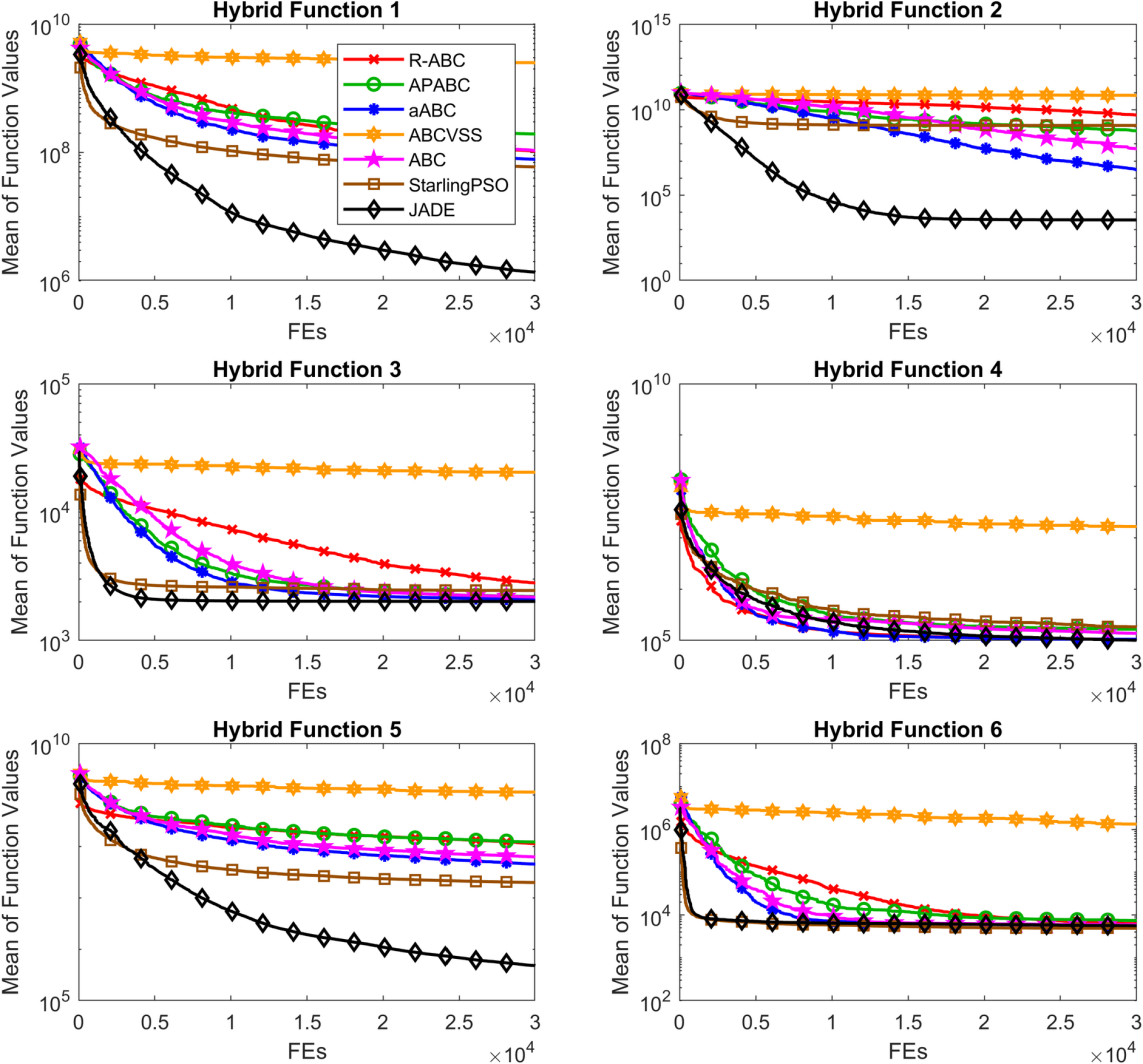
Convergence performance on the CEC2014’s hybrid functions with *D* = 100.

Table 11 shows the results on the CEC2014’s hybrid functions. Table 12 shows the results of the Wilcoxon’s rank sum test. Fig 15 shows the convergence performance of the CEC2014’s hybrid functions. The solutions of the R-ABC algorithm are significantly better than the Starling PSO, ABC, APABC, and ABCVSS algorithms on 1, 1, 3, and 6 functions, respectively. Overall, the R-ABC algorithm is beaten by the JADE and aABC algorithms on the hybrid functions. However, the solutions of the R-ABC algorithm are similar to those of the JADE and aABC algorithms, and significantly better than those of the ABC, APABC, ABCVSS, and Starling PSO algorithms on Hybrid Function 4.

**Table 11:** Results for CEC2014’s hybrid functions (*MFE* = 30000).

Function	Stat	R-ABC	APABC	aABC	ABCVSS	ABC	Starling PSO	JADE
Hybrid Function 1	Mean	1.05E+08	1.94E+08	7.81E+07	2.50E+09	1.08E+08	5.95E+07	**1.36E+06**
	Min	7.78E+07	1.08E+08	2.01E+07	3.03E+08	5.67E+07	1.40E+07	**5.49E+05**
	Max	1.36E+08	3.30E+08	1.54E+08	3.99E+09	1.58E+08	2.42E+08	**2.28E+06**
	SD	1.32E+07	5.99E+07	3.06E+07	1.01E+09	2.81E+07	4.93E+07	**5.15E+05**
	Sig		+	-	+	=	-	-
Hybrid Function 2	Mean	4.90E+09	5.87E+08	3.09E+06	6.87E+10	5.33E+07	1.21E+09	**3.61E+03**
	Min	3.01E+09	3.11E+08	1.28E+05	3.70E+10	2.96E+06	3.26E+03	**2.15E+03**
	Max	8.31E+09	1.24E+09	2.63E+07	8.97E+10	5.55E+08	9.82E+09	**8.66E+03**
	SD	1.46E+09	2.44E+08	5.97E+06	1.61E+10	1.25E+08	2.49E+09	**1.93E+03**
	Sig		-	-	+	-	-	-
Hybrid Function 3	Mean	2.81E+03	2.20E+03	2.10E+03	2.04E+04	2.18E+03	2.45E+03	**2.02E+03**
	Min	2.41E+03	2.10E+03	2.00E+03	7.16E+03	2.11E+03	2.04E+03	**2.00E+03**
	Max	3.32E+03	2.38E+03	2.17E+03	4.01E+04	2.29E+03	3.23E+03	**2.04E+03**
	SD	2.28E+02	6.25E+01	3.26E+01	7.93E+03	5.39E+01	3.68E+02	**1.25E+01**
	Sig		-	-	+	-	-	-
Hybrid Function 4	Mean	1.03E+05	1.65E+05	1.06E+05	1.65E+07	1.38E+05	1.84E+05	**1.01E+05**
	Min	8.72E+04	1.04E+05	5.60E+04	2.37E+06	6.52E+04	1.27E+05	**9.77E+03**
	Max	**1.36E+05**	2.45E+05	1.49E+05	6.49E+07	1.96E+05	3.41E+05	1.91E+05
	SD	**1.18E+04**	3.15E+04	2.20E+04	1.61E+07	3.57E+04	4.70E+04	3.63E+04
	Sig		+	=	+	+	+	=
Hybrid Function 5	Mean	1.14E+08	1.22E+08	4.55E+07	1.15E+09	6.29E+07	1.99E+07	**4.80E+05**
	Min	8.64E+07	7.47E+07	2.40E+07	3.65E+08	2.67E+07	6.43E+06	**6.37E+04**
	Max	1.36E+08	2.22E+08	1.42E+08	2.53E+09	1.18E+08	3.96E+07	**8.77E+05**
	SD	1.47E+07	3.37E+07	2.45E+07	5.36E+08	2.52E+07	9.38E+06	**2.24E+05**
	Sig		=	-	+	-	-	-
Hybrid Function 6	Mean	6.27E+03	7.44E+03	5.64E+03	1.33E+06	5.93E+03	**4.90E+03**	5.62E+03
	Min	5.64E+03	6.82E+03	4.78E+03	2.38E+05	5.06E+03	**4.11E+03**	5.13E+03
	Max	7.62E+03	8.09E+03	6.34E+03	2.88E+06	6.62E+03	**5.79E+03**	6.19E+03
	SD	4.52E+02	3.41E+02	4.13E+02	8.50E+05	4.70E+02	4.96E+02	**3.10E+02**
	Sig		+	-	+	-	-	**-**

**Table 12:** The result of Wilcoxon’s test of the algorithm on the CEC2014’s hybrid functions.

R-ABC vs	-	=	+
APABC	2	1	3
aABC	5	1	0
ABCVSS	0	0	6
ABC	4	1	1
Starling	5	0	1
JADE	5	1	0

### Analysis of the perturbation

In addition, we analysed the impact of the perturbation. The sum of the reinforcement values is always equal to 1, so the average reinforcement value is 1/*D*. When the number of dimensions is high, the magnitude of perturbation is probably narrow. A narrow magnitude of perturbation probably makes the performance of the R-ABC algorithm drop especially when the optimal value of each dimension is far from those of other dimensions. Therefore, two new parameters, *γ* and *κ*, are introduced to analyse the impact of perturbation. The parameter *γ* is used to increase the degree of reward and penalty. The value of *γ* is set to 1, 2, 3, 4, 5, 10, 15, and 20. Eqs (8) and (9) are replaced by Eqs (12) and (13), respectively.

If $Fit({\bar{v}}^{t}{}_{i})$ > $Fit({\bar{x}}^{t}{}_{i})$ then

$$r_{j}^{t+1}=\left\{ \begin{array}{l} r_{j}^{t}+\alpha \gamma \left(1-r_{j}^{t}\right),j=d\\ r_{j}^{t}\times \left(1-\alpha \gamma \right),j\neq d \end{array}\right.$$ (12)

If $Fit({\bar{v}}^{t}{}_{i})$ < = $Fit({\bar{x}}^{t}{}_{i})$ then

$$r_{j}^{t+1}=\left\{ \begin{array}{l} r_{j}^{t}\times \left(1-\beta \gamma \right),j=d\\ \frac{\beta \gamma }{D-1}+r_{j}^{t}\times \left(1-\beta \gamma \right),j\neq d \end{array}\right.$$ (13)

The parameter *κ* is used to magnify the reinforcement values. The value of *κ* is set to 1, 2, 3, and 4, respectively. Eq (11) is replaced by Eq (14).

$$v_{i,j}^{t}=x_{i,k}^{t}+\Phi_{i,j}^{t}\cdot r_{j}^{t}\cdot \kappa \cdot \left(x_{i,k}^{t}-x_{BSF,k}^{t}\right)$$ (14)

The analysis of perturbation was conducted on the CEC2005’s shifted functions for 100 dimensions. Note that it is the same as the original R-ABC algorithm when both *γ* and *κ* are set to 1.

**Fig 16:**
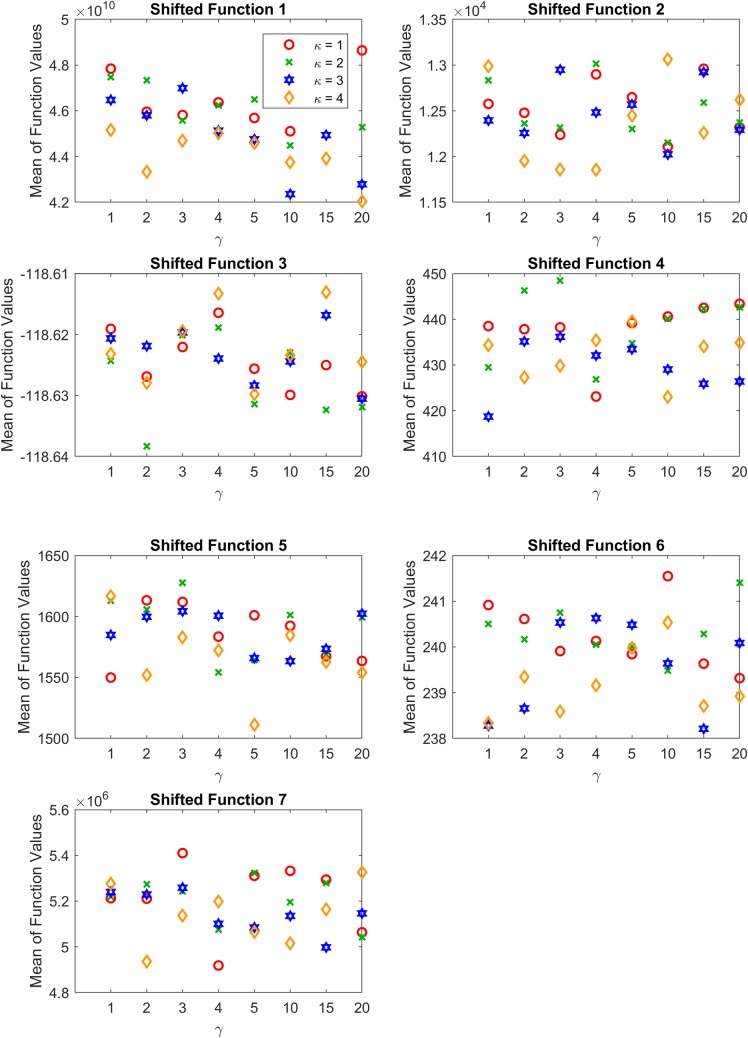
The final results of the CEC2005’s shifted functions with different values of *γ* and *κ*.

Fig 16 shows the final results of the CEC2005’s shifted functions with different values of *γ* and *κ*. There is no evidence that any value of *γ* gives solutions significantly better than other values. It means that multiplying the degree of reward and penalty by 1, 2, 3, 4, 5, 10, 15, and 20 does not significantly improve the performance of the R-ABC algorithm. Although there is no significant impact of *γ*, different values of *κ* give different final results. The Friedman’s test is used to evaluate the results from different values of *κ* as shown in Table 13. For the Shifted Function 1, 2, 5, 6, and 7, the best solutions are obtained when *κ* is set to 4. For the Shifted Function 3, the best solution is obtained when *κ* is set to 2. For the Shifted Function 4, the best solution is obtained when *κ* is set to 3.

The results of the experiment show that, in all cases, the original R-ABC algorithm (*γ* = 1 and *κ* = 1) does not give the best solution. The performance of the R-ABC algorithm can improve by changing the value of *κ*. Each benchmark function requires a different value of *κ* to obtain the best solution.

**Table 13:** Rankings by the Friedman’s test on the CEC2005’s shifted functions with different *κ*.

Function	*κ*			
	1	2	3	4
Shifted Function 1	2.7750	2.6937	2.2875	**2.2438**
Shifted Function 2	2.5187	2.5000	2.5187	**2.4625**
Shifted Function 3	2.5688	**2.3438**	2.5750	2.5125
Shifted Function 4	2.6500	2.6062	**2.3500**	2.3937
Shifted Function 5	2.5062	2.5562	2.5688	**2.3688**
Shifted Function 6	2.6687	2.5687	2.4687	**2.2938**
Shifted Function 7	2.6563	2.6312	2.4563	**2.2562**

## Conclusions

In this paper, we have integrated a reinforcement learning method for solution updates in the Artificial Bee Colony algorithm. We applied a positive-negative reinforcement to the dimensions of candidate food sources during the onlooker bee phase. All dimensions of a solution are updated at the same time, based on a corresponding reinforcement value. These reinforcement values indicate the range of the value in each dimension. A dimension which provides better solutions after a change will vary within a wider range during the update.

There are two differences between the original ABC and the R-ABC algorithms. First, different information is shared between employed bees and onlooker bees. Each employed bee in the R-ABC algorithm shares not only the quality of food source but also which dimension should be changed to find a better food source. Second, all dimensions of R-ABC are updated in every iteration while the original ABC algorithm updates only one dimension at a time. Generally, this will improve convergence speed for the R-ABC algorithm compared with the original ABC algorithm.

The reinforcement learning method enables an onlooker bee to update the values in all dimensions with different ranges according to the information from employed bees. This makes the ranges of update in each dimension adaptable to all kind of functions which results in making the algorithm capable of handling a wider range of real world problems.

The proposed algorithm was tested on eight basic numerical benchmark functions, seven CEC2005’s shifted functions, and six CEC2014’s hybrid functions. The selected basic numerical benchmark functions were chosen from four categories: unimodal separable functions, unimodal non-separable functions, multimodal separable functions, and multimodal non-separable functions. We tested the basic functions with 100, 500, 700 and 900 dimensions, the CEC2005’s shifted functions with 100, 500, 700 and 900 dimensions, and the CEC2014’s hybrid functions with 100 dimensions.

Compared with other algorithms, the R-ABC algorithm provides the best mean solutions for all basic benchmark functions with all tested dimensional size. Categories of benchmark functions do not consistently affect the solution quality of the R-ABC algorithm. Compared with other ABC variants on the CEC2005’s shifted functions, the results provide evidence that the R-ABC algorithm is less sensitive to the growing number of dimensions than some algorithms in the majority of cases. Compared with other algorithms on the CEC2014’s hybrid functions, the R-ABC algorithm is better than or at least comparable to the ABC variants.

In short, our results suggest that using reinforcement to differently control the degree of variation across dimensions in a high-dimensional problem is an effective modification to the original ABC. This technique provides good quality solutions to high-dimensional problems without sacrificing convergence speed. In the future, the value of parameter *κ* can be studied and modified to improve the performance of the R-ABC algorithm in the aspects of solution quality and convergence speed.

## Supporting information

S1 Appendix Proof of the reinforcement values’ sum. (PDF)
