# Supplementary material for: Reinforcement learning for solution updating in Artificial Bee Colony
Source: PLoS One. 2018 Jul 17;13(7):e0200738. doi: 10.1371/journal.pone.0200738 (PMC6049945; doi:10.1371/journal.pone.0200738)
Supplement: S1 Appendix — (PDF) [file pone.0200738.s001.pdf]

S1 Appendix

The sum of the reinforcement values after positive reinforcement is always equal to 1 as shown in the following proof.

$$\sum_{j=1}^{D} r_j^{t+1} = \left[r_d^t + \alpha\left(1 - r_d^t\right)\right] + \sum_{j=1,\, j\neq d}^{D} \left[r_j^t \times \left(1-\alpha\right)\right]$$

$$\sum_{j=1}^{D} r_j^{t+1} = r_d^t + \alpha - \alpha r_d^t + \left(1-\alpha\right) \times \sum_{j=1,\, j\neq d}^{D} r_j^t$$

$$\sum_{j=1}^{D} r_j^{t+1} = \alpha + \left(r_d^t - \alpha r_d^t\right) + \left(1-\alpha\right) \times \sum_{j=1,\, j\neq d}^{D} r_j^t$$

$$\sum_{j=1}^{D} r_j^{t+1} = \alpha + r_d^t\left(1-\alpha\right) + \left(1-\alpha\right) \times \sum_{j=1,\, j\neq d}^{D} r_j^t$$

$$\sum_{j=1}^{D} r_j^{t+1} = \alpha + \left(1-\alpha\right) \times \left(r_d^t + \sum_{j=1,\, j\neq d}^{D} r_j^t \right)$$

$$\sum_{j=1}^{D} r_j^{t+1} = \alpha + \left(1-\alpha\right) \times 1$$

$$\sum_{j=1}^{D} r_j^{t+1} = \alpha + 1 - \alpha$$

$$\sum_{j=1}^{D} r_j^{t+1} = 1$$

The sum of the reinforcement values after negative reinforcement is always equal to 1 as shown in the following proof.

$$\sum_{j=1}^{D} r_j^{t+1} = \left[r_d^t \times \left(1-\beta\right)\right] + \sum_{j=1,\, j\neq d}^{D} \left[\frac{\beta}{D-1} + r_j^t \times \left(1-\beta\right)\right]$$

$$\sum_{j=1}^{D} r_j^{t+1} = \left[r_d^t \times (1-\beta)\right] + \sum_{j=1,\, j\neq d}^{D} \left(\frac{\beta}{D-1}\right) + \sum_{j=1,\, j\neq d}^{D} \left(r_j^t \times (1-\beta)\right)$$

$$\sum_{j=1}^{D} r_j^{t+1} = \left[r_d^t \times (1-\beta)\right] + \beta + \left((1-\beta) \times \sum_{j=1,\, j\neq d}^{D} r_j^t\right)$$

$$\sum_{j=1}^{D} r_j^{t+1} = \beta + \left((1-\beta) \times \left(r_d^t + \sum_{j=1,\, j\neq d}^{D} r_j^t\right)\right)$$

$$\sum_{j=1}^{D} r_j^{t+1} = \beta + \left((1-\beta) \times 1\right)$$

$$\sum_{j=1}^{D} r_j^{t+1} = \beta + 1 - \beta$$

$$\sum_{j=1}^{D} r_j^{t+1} = 1$$
